# Regulation of the DNA Damage Response and Gene Expression by the Dot1L Histone Methyltransferase and the 53Bp1 Tumour Suppressor

**DOI:** 10.1371/journal.pone.0014714

**Published:** 2011-02-24

**Authors:** Jennifer FitzGerald, Sylvie Moureau, Paul Drogaris, Enda O'Connell, Nebiyu Abshiru, Alain Verreault, Pierre Thibault, Muriel Grenon, Noel F. Lowndes

**Affiliations:** 1 Genome Stability Laboratory, School of Natural Sciences, Centre for Chromosome Biology, National University of Ireland Galway, Galway, Ireland; 2 National Centre for Biomedical Engineering Sciences, National University of Ireland Galway, Galway, Ireland; 3 Institute for Research in Immunology and Cancer, Université de Montréal, Montréal, Québec, Canada; 4 Département de Pathologie et Biologie Cellulaire, Université de Montréal, Montréal, Québec, Canada; 5 Département de Chimie, Université de Montréal, Montréal, Québec, Canada; Ludwig-Maximilians-Universität München, Germany

## Abstract

**Background:**

Dot1L, a histone methyltransferase that targets histone H3 lysine 79 (H3K79), has been implicated in gene regulation and the DNA damage response although its functions in these processes remain poorly defined.

**Methodology/Principal Findings:**

Using the chicken DT40 model system, we generated cells in which the *Dot1L* gene is disrupted to examine the function and focal recruitment of the 53Bp1 DNA damage response protein. Detailed kinetic and dose response assays demonstrate that, despite the absence of H3K79 methylation demonstrated by mass spectrometry, 53Bp1 focal recruitment is not compromised in these cells. We also describe, for the first time, the phenotypes of a cell line lacking both *Dot1L* and *53Bp1*. *Dot1L^−/−^* and wild type cells are equally resistant to ionising radiation, whereas *53Bp1^−/−^/Dot1L^−/−^* cells display a striking DNA damage resistance phenotype. *Dot1L* and *53Bp1* also affect the expression of many genes. Loss of Dot1L activity dramatically alters the mRNA levels of over 1200 genes involved in diverse biological functions. These results, combined with the previously reported list of differentially expressed genes in mouse ES cells knocked down for *Dot1L*, demonstrates surprising cell type and species conservation of *Dot1L*-dependent gene expression. In *53Bp1^−/−^* cells, over 300 genes, many with functions in immune responses and apoptosis, were differentially expressed. To date, this is the first global analysis of gene expression in a *53Bp1*-deficient cell line.

**Conclusions/Significance:**

Taken together, our results uncover a negative role for Dot1L and H3K79 methylation in the DNA damage response in the absence of 53Bp1. They also enlighten the roles of Dot1L and 53Bp1 in gene expression and the control of DNA double-strand repair pathways in the context of chromatin.

## Introduction

In eukaryotic cells, DNA is present in a DNA-protein complex known as chromatin [1]. The building block of chromatin is the nucleosome. This structure consists of 147 bp of DNA wrapped around a histone octamer that contains two molecules each of the histones H2A, H2B, H3, and H4 [1], [2]. Chromatin is the physiological template for all DNA metabolic processes and chromatin structure is modulated via a number of mechanisms to regulate access to DNA [3]. These mechanisms include ATP-dependent chromatin remodelling [4], incorporation of histone variants [5] and several covalent modifications of histone residues (methylation, acetylation, phosphorylation, ubiquitination and sumoylation) [3].

Histone methylation occurs at a number of lysine and arginine residues [6]. One such residue is lysine 79 of histone H3 (H3K79). This modification was first identified in budding yeast and is conserved from budding yeast to human [7], [8], [9], [10], [11]. H3K79 methylation is catalysed by the enzyme DOT1L/KMT4, which adds one, two, or three methyl groups to generate H3K79me1, me2, and me3 respectively [7], [8], [12], [13]. The enzyme exhibits three distinctive features compared to other histone methyltransferases. Firstly, it displays histone methyltransferase (HMTase) activity exclusively towards histone H3 that is incorporated into nucleosomes, but not free H3 [8], [12], [13]. Secondly, its substrate, H3K79, is present in the α1 helix of the histone H3 globular core, unlike most well characterised histone modifications which occur on the exposed histone tails [3]. Thirdly, DOT1L is the only known lysine HMTase that does not possess a SET domain [14]. Instead, it contains an *S*-adenosyl methionine (SAM) binding domain similar to those found in arginine methyltransferases [15]. Mutation or deletion of this domain in DOT1L completely abrogates its histone methyltransferase activity [7], [8], [13], [16].

H3K79 methylation is involved in DNA metabolic processes such as the regulation of gene expression and the response to DNA damage in both yeast and higher eukaryotes. Cells are constantly exposed to a barrage of both exogenous and endogenous genotoxic agents that result in DNA damage [17]. To overcome the deleterious effects of this damage, cells have evolved complex signalling networks to detect and repair DNA damage before the cell divides [17]. These DNA damage response pathways play major roles in the maintenance of genomic integrity and tumour suppression.

53BP1 is a tumour suppressor protein that functions in checkpoint activation and DNA repair [18], [19]. The protein provides a direct link between chromatin and the DNA damage response, as 53BP1 is recruited specifically to sites of DNA damage through binding of its tandem Tudor domains to methylated histone residues. Histone-mediated recruitment of 53BP1 remains controversial. One of the residues implicated in this recruitment is methylated H3K79 [20]. Indeed, abrogation of H3K79me through siRNA knockdown of *DOT1L* in human cells has been reported to completely abolish 53BP1 focal recruitment [20]. However, contrary to these results, others have reported that H3K79 methylation is not involved in this process [21]. Instead, these studies concluded that 53BP1 binds directly to dimethylated histone H4 lysine 20 (H4K20me2) [21], [22], [23]. However, none of these studies completely excluded the possibility that both H3K79me and H4K20me might be involved in the recruitment of 53BP1. Further work is therefore important to assess the relative contributions of H3K79 and H4K20 methylation to 53BP1 recruitment at sites of DNA damage.

Interestingly, functions for both of these methylated histone residues in the DNA damage response have been conserved in evolution. In budding yeast, which does not possess H4K20 methylation [24], H3K79 methylation is required for activation of the 53BP1-related protein Rad9 and its mobilisation to sites of DNA damage [25], [26], [27]. Conversely, in fission yeast, which lacks a DOT1L homologue and H3K79 methylation [28], recruitment of the 53BP1-related protein Crb2 to regions of DNA damage occurs, at least in part, via binding to methylated H4K20 [28], [29].

DOT1L and H3K79 methylation also affect gene expression. In wild-type budding yeast, H3K79 methylation is extremely abundant (about 90% of H3 molecules are K79-methylated) and present throughout euchromatin, but is conspicuously absent from heterochromatic regions such as the silent mating type loci and sub-telomeric regions [13], [30]. The rationale behind this localisation pattern is that H3K79 methylation prevents inappropriate binding of the heterochromatic SIR proteins to euchromatic loci [13]. Consequently, budding yeast cells that lack H3K79 methylation or overexpress Dot1 cannot confine SIR proteins to heterochromatin, which leads to silencing defects of sub-telomeric and silent mating type reporter genes [8], [11], [12], [13], [30], [31], [32], [33], [34]. An additional consequence of SIR protein dissociation from heterochromatin in cells lacking H3K79 methylation is that they are now able to roam free throughout the nucleus and potentially interfere with expression of euchromatic genes.

Although much less abundant than in yeast cells (see Results), H3K79 methylation also influences heterochromatin structure in higher eukaryotes [35]. Surprisingly, despite the fact that Dot1L specifically methylates H3K79, the abundance of heterochromatin-specific histone marks, such as H4K20me3, is reduced in *Dot1L*-deficient mouse ES cells [35]. In addition, hypermethylation of H3K79 at the mouse *HoxA9* locus increases transcription of this gene [36]. In contrast, at the mouse *EnaCα* locus, increased levels of H3K79 methylation actually reduce gene expression [37]. Therefore, it does appear that the influence of H3K79 methylation on gene expression in vertebrate cells is locus-dependent. In vertebrates, it is possible that the impact of H3K79 methylation on gene expression depends on ill-defined aspects of the surrounding chromatin environment [9].

Clearly, the functions of H3K79 methylation in the DNA damage response and gene expression are far from resolved and this is particularly true in vertebrate cells. To further our understanding of the role of the Dot1 HMTase in these processes, we generated novel vertebrate cell lines lacking Dot1L and H3K79 methylation. We used the DT40 cell line derived from chicken lymphocytes [38], [39]. These cells present a number of advantages that make them an invaluable model system for genetic studies in vertebrate cells. Of particular relevance here, is their high frequency of homologous recombination (HR), which facilitates gene targeting and the generation of cell lines with specific gene knockouts. In addition, studies of chicken DT40 cell lines do not suffer from drawbacks of mouse or human cell lines, such as off-targets effects and incomplete sh/siRNA-mediated mRNA knockdown or contamination of ES cells by feeder cells. Here, we exploited a novel DT40 *Dot1L^−/−^* cell line to conduct detailed kinetic and DNA damage dose response assays not previously performed in other model systems. We demonstrate that 53Bp1 recruitment to sites of DNA damage caused by ionising radiation is not compromised in cells lacking Dot1L. For the first time, we also report studies of cells deficient in both 53Bp1 and Dot1L. Surprisingly, although abrogation of Dot1L function alone does not alter survival following DNA damage, *53Bp1^−/−^/Dot1L^−/−^* double mutant cells display a striking resistance to ionising radiation. Microarray analysis of *53Bp1^−/−^* and *Dot1L^−/−^* cell lines shows that 53Bp1 and Dot1L both affect gene expression and that *Dot1L^−/−^* cells exhibit dramatically altered expression of many RNAs.

## Materials and Methods

### Cell lines

**Figure 1:**
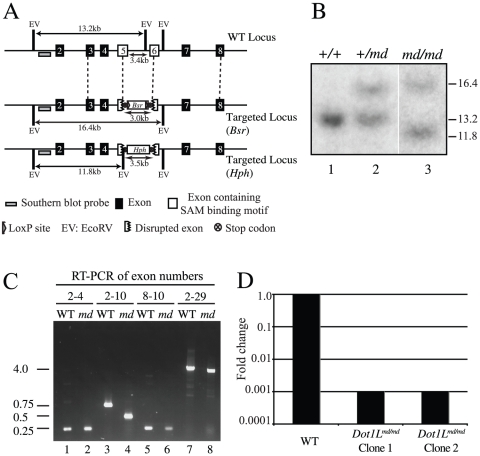
Generation of *Dot1L* deficient cell lines. A. Schematic of the strategy to disrupt the chicken *Dot1L* SAM binding domain and confirm the deletion by Southern blotting. Exons are numbered. *Bsr*: blasticidin resistance gene; *Hph*: hygromycin resistance gene. EV: *Eco*RV restriction sites. Grey rectangle: probe for Southern blots. The figure is approximately drawn to scale. B. Southern blot to confirm targeted integration of the *Dot1L* disruption constructs. Digestion of the WT *Dot1L* locus with *Eco*RV generates a band of 13.2 kb when hybridised with the probe shown in A. Following targeted integration of the blasticidin construct, the size of the band increases to 16.4 kb. Targeted integration of the hygromycin construct results in a band of 11.4 kb. C. Reverse transcription PCR of the indicated exons of *Dot1L* in WT and *Dot1L^md/md ^*cells. D. RT-qPCR to quantify the levels of *Dot1L* RNA in WT and *Dot1L^md/md^* cell lines.

Cell lines were created in the DT40 wild-type background, also known as WT18. *Dot1L^−/−^* and *53Bp1^−/−^Dot1L^−/−^* cell lines were also generated in the Cre1-DT40 WT cell line [40], [41] to facilitate future generation of multiple knockout cell lines. Cre1-DT40 WT cells express the Cre1 recombinase that can translocate into the nucleus in response to 4-hydroxytamoxifen. This recombinase [42] will recognise tandem repeats of *LoxP* sites and allow the removal of DNA located between the two repeats. This property allows multiple use of the same selective cassette surrounded by *LoxP* sites (Figure 1A). Note that the Cre1-DT40 WT cell line has some limitations including a defective response to nocodazole and an ionizing radiation-induced centrosome amplification phenotype [43]. Importantly, Cre1-DT40 WT cells display the same survival rate [43] and γH2AX/53Bp1 focal accumulation as WT18 cells (Figures S6 and S7). All experiments shown in the manuscript were performed with cell lines in the WT18 background. The only exception was the quantification of γH2AX and 53Bp1 foci formation, which were performed in Cre1-DT40 WT and Cre1-DT40 *Dot1L^−/−^* cells in comparison with WT18 cells (Figures 4, S6 and S7). However, similar to the Cre1-DT40 *Dot1L^−/−^* cells, both γH2AX and 53Bp1 foci were obtained in the WT18 *Dot1L^−/−^* cell line (data not shown).

### Cell culture

DT40 cell lines were cultured at 39.5°C and 5.0% CO_2_ under humidified conditions in RPMI 1640 (with L-glutamine and sodium bicarbonate), 10% foetal bovine serum (FBS), 1% chicken serum, 100 U/ml penicillin, and 100 µg/ml streptomycin. Media and supplements were obtained from either Sigma (Dublin, Ireland) or Lonza (Berkshire, UK).

### Construction of targeting vectors

The sequence of the chicken *Dot1L* locus was obtained from the NCBI (NM_032482.1) and Ensembl (ENSGALG00000000843) chicken genome databases. The *Dot1L* targeting vectors pLoxBLASTDot1L, pLoxNEODot1L, and pBSHygroDot1L were constructed to delete 3.4 kb of genomic sequence encompassing exons 5 and 6 (Figure 1A). A 3.8 kb region present upstream of the targeted site was amplified from genomic DNA by PCR using the forward primer 5′-GACGGTACCTGACTAGCTAAATCCCAGATCTCAAGCTTGCTATGG-3′, which contained a *Kpn*I site and the reverse primer 5′-GACGTCGACTTAGCTAGTCAGTGTTCAGCTTCATCGGTTGGGTG-3′, which contained a *Sal*I site. This PCR product was inserted, as a *Kpn*I-*Sal*I fragment, upstream of the resistance cassettes of the pLOXBLAST, pLOXNEO [41] and pBSHygro plasmids. Similarly, a 5.8 kb region downstream of the targeted site was amplified from genomic DNA by PCR using the forward primer 5′-GACACTAGTTGACTAGCTAACCGCAGCCACGAACTGCAAACATC-3′, which contained an *Spe*I site and the reverse primer 5′-GACGCGGCCGCTGACTAGCTAAGCACGGCGATGCCCATTACTGC-3′, which contained a *Not*I site. This PCR product was inserted as an *Spe*I-*Not*I fragment downstream of the resistance cassettes of the targeting vectors. Primers also contained stop codons in all six reading frames. Transfection was performed by electroporation of *Kpn*I-linearised targeting constructs using the Gene Pulsar electroporation apparatus (Bio-Rad, Wicklow, Ireland). Southern blotting was carried out using a probe external to the targeting construct. The probe was labeled with either [α-^32^P] or digoxigenin (PCR Dig probe synthesis kit, Roche, Germany) and amplified using the forward primer Dot1paprobeF 5′-GAGCCTATACCCTTCTGACACTTG-3′ and the reverse primer Dot1paprobeR 5′-GCACTGCAATCACGC TTGTAAGAC-3′.

Transient transfections were performed using pcDNA.hDot1L, containing the human *DOT1L* cDNA under the control of the CMV promoter (a kind gift from Dr. Yi Zhang, UNC Chapel Hill, USA), endotoxin-free circular plasmids and the Amaxa cell line nucleofection kit T (Lonza, Berkshire, UK), as described in the manufacturers' protocol.

### Proliferation and clonogenic survival analysis

For cell proliferation analysis, cultures were seeded in triplicate at equal cell densities (1×10^4^ cells/ml or 1×10^5^ cells/ml) and counted using a haemocytometer every 24 hours. Survival of cells following ionizing radiation was determined by clonogenic survival assays in methylcellulose medium after exposure to a ^137^Cs irradiator (Mainance Engineering Ltd, UK) at a dose rate of 21.57 Gy/minute. Colonies formed by surviving cells were counted 7–10 days later.

### Peptides, antibodies and recombinant H3

Western blotting was performed using antibodies recognising monomethylated (anti-H3K79me1, ab2886, Abcam), dimethylated (anti-H3K79me2, ab3594, Abcam), and trimethylated (anti-H3K79me3, ab2621, Abcam) lysine 79 of histone H3, or a C-terminal peptide of H3 (anti-H3, ab1791, Abcam). H3K79-methylated peptide (H3K79me1 - ab4555, H3K79me2 - ab4556, Abcam) concentrations were assessed by measuring their absorbance at 205nm. H4K20-methylated peptide (H4K20me0 – ab14963, H4K20me1 – ab17043, H4K20me2 – ab14964, H4K20me3 – ab17567) concentrations were determined by measuring their absorbance at 230 nm in a Nanodrop spectrophotometer. Recombinant histone H3 was a generous gift from Dr. Andrew Flaus.

### Immunofluorescence microscopy

Cells were irradiated or mock-treated and adhered to poly-L-lysine coated slides before fixation in 4% paraformaldehyde. Cells were permeabilised in 0.125% Triton-X-100 and non-specific antibody binding blocked with 1% BSA. Cells were then incubated with affinity purified anti-chicken 53Bp1 [44] or anti-γH2AX mouse monoclonal antibodies (Millipore). FITC- and Texas Red-conjugated secondary antibodies (Jackson Immunoresearch) were used. Images were acquired using the Olympus BX51 microscope and Openlab software (Improvision). 0.4 µm Z-stacks were collected and merged. All foci and cell percentages were determined by blind-counting 50–100 cells per sample.

### Histone preparation and protein digestion

Histones from DT40 cells were prepared from 10^7^ cells and digested as previously described [45]. The quality of histone preparations (2–4 mg/ml of the 4 equimolar core histones) and the purification yield (45–65%) were evaluated on a Coomassie-stained gel (not shown). Intact core histones (approximately 10 µg of acid-extracted total protein) were separated using an Agilent 1100 HPLC system equipped with a micro-fraction collector. Separations were performed on a microbore Biobasic C_18_ column (5 µm, 300Å), 150×1 mm i.d., with a solvent system consisting of 0.1% TFA in water (v/v) (solvent A) and 0.1% TFA in acetonitrile (v/v) (solvent B). Gradient elution was performed from 5–70% B in 60 minutes at 15 µl/min. Fractions were collected in a 96-well plate in 30 second time slices. The fractions containing each core histone peak were pooled together. Fractions were dried in a Speed-Vac. Proteins were resuspended in 0.1 M ammonium bicarbonate (without pH adjustment). This protein solution was derivatized in a 1:1 volume ratio using propionic anhydride. After a 30 minutes at ambient temperature, samples were dried a second time, resuspended in 0.1 M ammonium bicarbonate, and digested overnight at 37°C using 1 µg of trypsin. Samples were acidified with 5% TFA in water (v/v) prior to mass spectrometry (MS) analysis. Based on the fact that peptides generated by trypsin cleavage of non-methylated or mono-methylated H3K79 or H4K20 were observed in much lower amounts than the propionylated peptides, we conclude that propionylation of H3K79 and H4K20 nearly went to completion. This is an important control because a low efficiency of propionylation would lead to an overestimation of the abundance of di- and tri-methylation relative to non-methylated and mono-methylated molecules. Without propionylation, the latter would be cut by trypsin after H3K79 and H4K20 and, therefore, excluded from our analysis.

### Global LC-MS/MS analysis of histone H3 fractions

Histone H3 tryptic digests were injected onto a Thermo Electron LTQ-Orbitrap XL mass spectrometer equipped with a nano electrospray interface. The instrument was operated in positive ion mode with a one-second survey scan from *m/z* 300 to 1600 at 60 000 resolution. The survey scan was followed by MS/MS in data dependent mode on the three most intense precursor ions within the LTQ ion trap. Liquid chromatography was performed using an Eksigent nano LC system. The trapping (4cm×360 µm i.d.) and analytical columns (10cm×150 µm i.d.) were packed in-house using teflon fused silica tubing and Phenomenex Jupiter C_18_ (5 µm, 300Å) material. The solvent system consisted of 0.2% formic acid in water (v/v) (solvent A) and 0.2% formic acid in acetonitrile (v/v) (solvent B). Gradient elution was performed from 0–50% B in 60 minutes at 600 nL/min. Raw LC-MS data files were converted to mgf file format and then submitted to Mascot for protein identification.

### Global FAIMS-MS/MS analysis of histone H4 fractions

Histone H4 tryptic digests were dried and resuspended in 50:50 methanol:water containing 0.2% formic acid (v/v). The Eksigent nano LC separation module was removed and replaced by a FAIMS module. Samples were introduced into the FAIMS instrument by direct infusion using a syringe pump operated at 600 nL/min. The LTQ-Orbitrap XL mass spectrometer was fitted with a nano electrospray interface. The instrument was operated in positive ion mode, and programmed to perform full scan acquisition from *m/z* 200 to 1600 while ramping the FAIMS compensation voltage (CV) from −50 to 0 V. Tandem MS was performed on a targeted list of histone H4 tryptic peptides for sequence confirmation.

### Targeted LC-MS/MS analysis of histone H3 fractions using MRM

To determine their abundance accurately, specific histone H3 tryptic peptides were quantified by multiple reaction monitoring (MRM) [46] using the MIDAS strategy on an AB/MDS Analytical Technologies 4000 Q-trap hybrid triple quadrupole-linear ion trap mass spectrometer equipped with a Nanospray II interface. Separations were performed using an Agilent 1100 HPLC system with dynamic flow splitting. All columns and solvents were identical to those listed under “global LC-MS/MS analysis of histone H3 fractions”. Chicken histone H3 sequences from the Swiss-Prot database were imported into the MRM pilot software. The 4 most intense *b* and/or *y* fragment ions were chosen to generate theoretical MRM transitions in the Analyst software. A ten-millisecond dwell time was used to monitor the MRM transitions. The MRM experiment was followed by an enhanced product ion (EPI) scan in IDA mode to confirm the sequences of the detected peptides.

### Reverse transcription PCR

RNA was extracted from cells using TriReagent (Invitrogen, Paisley, UK). A cDNA pool was generated by reverse transcription using the Superscript First-Strand Synthesis kit (Invitrogen, Paisley, UK). Reverse transcription (RT) was primed using oligo-dT primers according to the manufacturer's protocols. Primers used to amplify *Dot1L* cDNA fragments were: Exon2F 5′ GACAAACACCATGATGCTGCTCATG-3′, Exon4R 5′-CTGATGGATGCTGTCGATGGCG-3′, Exon8F 5′-GAGGTGACTTCCTCTCAGAAGAATGG-3′, Exon10R 5′-CGACTGTTAATTCTGAAATTCAGAGGTG-3′.

### Microarray analysis

Total RNA samples were prepared using TriReagent (Invitrogen, Paisley, UK), subjected to DNaseI digestion and further purified using Qiagen RNAEasy columns (Qiagen, West Sussex, UK). RNA quality was confirmed using the Agilent 2100 Bioanalyzer and spectrophotometric methods. RNA was amplified using the NuGen Ovation RNA Amplification System V2, fragmented and labelled using the FL-Ovation cDNA Biotin Module V2 (NuGen, San Carlos, CA, USA), and hybridised to the Affimetrix GeneChip chicken genome microarray in accordance with NuGen guidelines. Array hybridisation was performed in triplicate using three independent RNA samples from each cell line. Raw data excel files were imported into GeneSpring GX 7.3 (Agilent Technologies) using RMA preprocessing, before per-chip (normalised to 50^th^ percentile) and per-gene (normalised to median signal) normalisations were performed. Non-expressed genes were removed by filtering on control signal using the cross-gene error model. Genes whose differential expression was significant were identified using Welch's approximate t-test, with a Benjamini and Hochberg false discovery rate of 5% and a fold change cutoff of 1.5.

All microarray data is MIAME compliant, as detailed on the MGED Society website http://www.mged.org/Workgroups/MIAME/miame.html. The raw data has been deposited in the MIAME compliant database ArrayExpress (http://www.ebi.ac.uk/microarray-as/ae/) under accession numbers E-MEXP-2721 (WT clone18 vs *53Bp1^−/−^* comparison) and E-MEXP-2722 (WT clone18 vs *Dot1L^−/−^* comparison). Data have been released in the public domain upon acceptance of this manuscript.

### Reverse transcription followed by quantitative real-time PCR

RNA samples were prepared from cells using TriReagent (Invitrogen, Paisley, UK). Quantitative real-time PCR was performed using the Applied Biosciences 7500-Fast system and the Applied Biosystems real-time PCR Sybr Green master mix (Applied Biosystems, Warrington, UK). All reactions were performed in triplicate. The complete list of primers used is provided in Supplementary Table S1.

### Fluorescence Activated Flow Cytometry (FACS)

To analyse DNA content, cells were fixed in 70% ethanol at −20°C. Cells were then stained using propidium iodide (Sigma, Dublin, Ireland), and analysed using the FACS Calibur platform and CellQuest software (BD Biosciences, Belgium).

## Results

### Generation of the *Dot1L^md/md^* cell line

The SAM binding domain is essential for the HMTase activity of both budding yeast Dot1 and human DOT1L [7], [8], [13], [16]. This fact was exploited to generate a cell line lacking Dot1L activity. The SAM binding domain is encoded by exons 5, 6, and 9. We designed a strategy to disrupt exons 5 and 6 by deleting a portion of these exons and integrating in-frame stop codons into both exons to disrupt translation of downstream sequences (Figure 1A). Integration of the resistance cassette should also perturb the reading frame of downstream sequences. In total, 3.4 kb of genomic sequence, corresponding to 245 RNA nucleotides or 83 amino acids, were deleted from the *Dot1L* gene. Our gene targeting strategy was predicted to generate a cell line, initially termed *Dot1L^md/md^*, in which Dot1L is methyltransferase deficient.

Targeting was performed in two DT40 wild-type backgrounds, WT18 [38] and Cre1-DT40, in which the Cre recombinase is stably integrated (see cell lines section of material and methods) [40]. In addition, targeting was also carried out in *53Bp1^−/−^* cell lines generated in the WT18 [44] and Cre1-DT40 (data not shown) backgrounds. Phenotypic analysis was carried out in both sets of cell lines (see cell lines section of material and methods). With the exception of the quantification of foci, which was performed in Cre1-DT40 backgrounds (Figures 4, S6 and S7), results obtained in the DT40 WT18 background cell lines are presented here.

Drug-resistant clones were screened by Southern blotting to identify targeted integration events (Figure 1B). As expected, a single band of 13 kb was detected in WT cells (lane 1). Heterozygous *Dot1L^+/md^* clones generated following transfection with the neomycin knockout construct were identified by detection of two bands: the WT band of 13 kb, and a 16 kb band corresponding to the targeted allele (lane 2). Integration of the hygromycin knockout construct at the remaining WT locus was demonstrated by the disappearance of the 13kb WT band, and the appearance of a correctly targeted 12 kb band (lane 3).

Reverse transcription PCR (RT-PCR) was performed to characterise *Dot1L* expression in *Dot1L^md/md^* cells (Figure 1C). Primers were designed to amplify the exons indicated in Figure 1C. As expected, expression of exons 2–4 was detected in both WT and *Dot1L^md/md^* cell lines (lanes 1 and 2). Transcription of exons downstream of the targeted integration site was detected in *Dot1L^md/md^* cells (lanes 5 and 6). In addition, transcription of exons 2 to 10, encompassing the targeted exons 5 and 6, was also detected in WT and *Dot1L^md/md^* cells, although the latter produced a shorter product (lanes 3 and 4). Sequencing of the RT-PCR products demonstrated that the *Dot1L* mRNA from *Dot1L^md/md^* cells contained exons 2–4 and exons 7–10, but lacked all of exons 5 and 6. Indeed it was possible to amplify the *Dot1L* transcript from exons 2 to 29 from *Dot1L^md/md^* cells (lane 8) suggesting the possibility of in-frame translation of sequences located upstream and downstream of the targeted region. In conclusion, a splicing event of exons 5 and 6 was generated by the introduction of the targeted construct.

To determine the level of *Dot1L* expression in our *Dot1L^md/md^* cells, we analysed *Dot1L* RNA levels in a quantitative manner using RT-PCR. Expression of *Dot1L* sequences downstream of the targeted site was reduced roughly 1000-fold in two independent *Dot1L^md/md^* cell lines (Figure 1D). Thus, not only had our gene targeting strategy successfully removed exons 5 and 6, which encode the SAM binding domain, it also severely reduced expression of a *Dot1L* mRNA containing an internal stop codon. Therefore, we re-designated our *Dot1L^md/md^* cells as *Dot1L^−/−^* cells to indicate defective gene function.

### H3K79 methylation in *Dot1L^−/−^* cells

**Figure 2:**
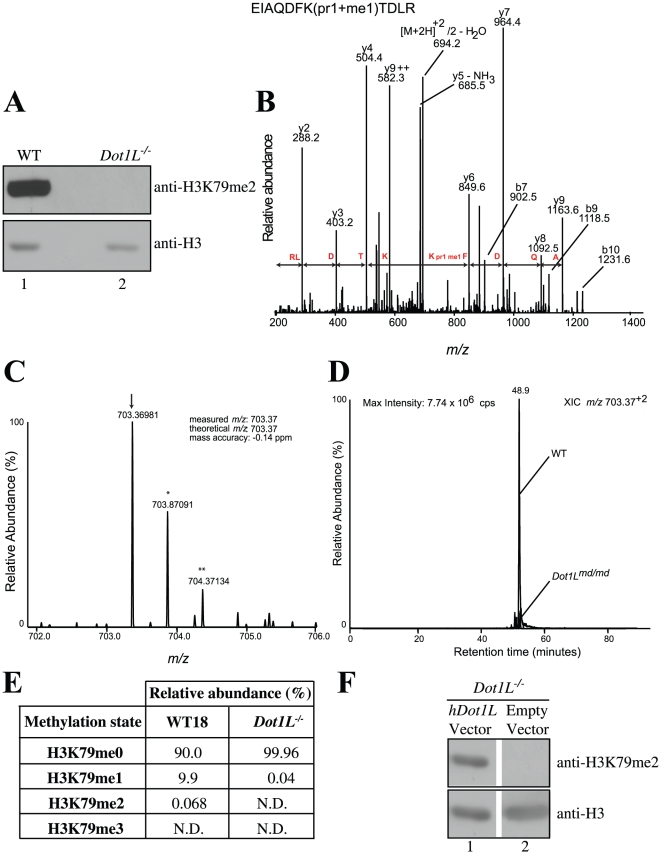
Histone H3 Lysine 79 is not methylated in *Dot1L^−/−^* cells. A. Western blot showing the loss of H3K79 dimethylation in *Dot1L^−/−^* cells. The membrane was stripped and reprobed with an antibody against total H3 to demonstrate equivalent loading. B. Collision-induced fragmentation spectrum of precursor ion *m/z* 706.37^2+^ (shown in C). The spectrum shows a nearly complete *y*-ion series. The mass difference between the *y*4 and y6-ion confirms the presence of a single propionyl and methyl group on H3K79 (pr1+me1). C. Doubly charged precursor ion with the measured *m/z* ratio expected for peptide EIAQDFK_79(pr1+me1)_TDLR. The monoisotopic peak containing only ^12^C atoms (arrow) and those containing a single ^13^C (*) or two naturally occurring ^13^C atoms (**) are shown. D. Extracted ion chromatograms (XIC) showing the abundance of the doubly charged precursor peptide containing H3K79me1 (pr1+me1) in WT and *Dot1L^−/−^* cells. E. Relative abundance of the various H3K79 methylation states determined by MRM mass spectrometry in total histones isolated from WT and *Dot1L^−/−^* cells. ND: not detected. F. Western blot showing that the absence of H3K79me2 in *Dot1L^−/−^* cells can be complemented by transient transfection of plasmid containing the human *Dot1L* cDNA.

To investigate whether the *Dot1L^−/−^* cell lines were indeed deficient in Dot1L HMTase activity, the level of H3K79 dimethylation was determined by western blotting using an antibody supposedly directed against H3K79me2. While H3K79 methylation was readily detected in WT cells (Figure 2A, lane 1), no methylation of this residue was detected in *Dot1L^−/−^* cells (lane 2), even after blot overexposure. Note that this antibody also recognises H3K79me1 to a lesser extent (Figure S1B and see next section). We also attempted to perform western blots for H3K79 mono- and trimethylation. However, the antibody against H3K79me1 cross-reacted with unmodified recombinant histone H3 (Figure S1A), and H3K79me3 could not be detected in WT or *Dot1L^−/−^* cells (data not shown).

Mass spectrometry (MS) was performed to determine the impact of *Dot1L* gene disruption on H3K79 mono-, di-, and trimethylation. Histones were acid-extracted from asynchronous populations of wild-type (WT) and *Dot1L^−/−^* cells. Intact core histones were further purified by reversed-phase HPLC. Purified H3 was propionylated to block trypsin digestion at non-modified and mono-methylated K79. After propionylation (pr), trypsin digestion generates four peptides of the same length that include K79. These peptides correspond to the unmodified (me0 pr1), mono- (me1 pr1), di- (me2 pr0) and tri-methylated (me3 pr0) forms of H3K79. They can all be readily distinguished based on their mass-to-charge ratio. After propionylation, tryptic digests were analysed by nanoLC-MS/MS. The fractions of total H3 that are mono-, di- or tri-methylated at K79 was quantified by multiple reaction monitoring [46] and are shown in Figure 2E. The fragmentation and precursor ion spectra of the propionylated peptide containing H3K79me1 (me1 pr1) are shown in Figures 2B and 2C, respectively. Mono-methylation (me1 pr1) of H3K79 was by far the most abundant methylated species in WT cells where it represents approximately 10% of total H3 (Figure 2E). This is a substantial figure which implies that, on average, an H3K79me1 molecule is present every five nucleosomes. Intriguingly, using either product ion scans or MRM, H3K79me2 was present in low amounts (not shown in Figure 2) and H3K79me3 was not detected in total histones prepared from wild-type DT40 cells. The low amount of H3K79me2 present was surprising given the strong H3K79me2 signal detected by western blotting (Figure 2A). However, using a peptide competition experiment we found that the antibody used here (Figure 2A) is not entirely specific to dimethylated H3K79 and probably cross-reacts with this residue when it is monomethylated (Figure S1B). This likely explains the strong signal detected in Figure 2A since, based on MS, the monomethylated form is present at nearly 150-fold greater abundance than the dimethylated form (Figure 2E). The MRM search list included precursor ions corresponding to the four modified states of H3K79 described above. Although MRM is very sensitive [45], it failed to identify ions that contained H3K79me3 in spite of the fact that the ions corresponding to unmethylated (pr1) and mono-methylated K79 (me1 pr1) were highly abundant (data not shown). We therefore conclude that H3K79me3 is either absent or present in very small amounts in wild-type DT40 cells. This is not unique to chicken DT40 cells; similar results were previously reported in total histones isolated from asynchronous populations of HeLa [47] and mouse ES cells [35]. In *Dot1L^−/−^* cells, essentially all H3K79me2 was absent (Figure 2E) and only a very small amount of H3K79me1 remained (Figures 2D and E).

### Human DOT1L complements Dot1L deficiency in chicken DT40 cells

To prove that the absence of H3K79 methylation was due to disruption of *Dot1L*, an expression vector containing a *hDOT1L* cDNA [7] was transiently transfected into *Dot1L^−/−^* cells. Due to the high level of conservation between human and chicken histone H3 and Dot1L (Figure S2), we predicted that human DOT1L would methylate H3K79 in chicken cells. Indeed, hDOT1L was previously found to methylate chicken nucleosomes *in vitro* [16]. As shown in Figure 2F, transfection of the *hDOT1L* expression construct into DT40 *Dot1L^−/−^* cells resulted in an increase in H3K79 methylation (lane 1) compared to transfection with the corresponding empty vector (lane 2).

### Dot1L deficiency does not affect histone H4 lysine 20 methylation

Both H3K79 and H4K20 methylation (H4K20me) have been implicated in the DNA damage response [20], [21], [22], [25], [29]. Because of this, we sought to determine whether disruption of the *Dot1L* gene had any influence on global levels of H4K20me. This was technically challenging because the short tryptic peptide containing H4K20 (20- KVLR -23) did not bind to our reversed-phase HPLC column even after propionylation. To circumvent this retention issue, we used an MS strategy that bypasses the need to separate tryptic peptides by reversed-phase HPLC prior to their detection in the mass spectrometer. This approach, known as high-field asymmetric waveform ion mobility spectrometry (FAIMS), separates precursor ions in the gas phase [48]. Histone H4 was propionylated to prevent cleavage at unmodified or mono-methylated lysine 20, digested with trypsin, and the resulting peptides were infused into a FAIMS unit coupled to an Orbitrap mass spectrometer. Using FAIMS, we readily detected a doubly charged precursor ion with *m/z* 272.20 corresponding to a dimethylated form of the H4 peptide (20- K_me2_VLR -23) (Figure S3). The fragmentation spectrum of this precursor ion confirmed its identity and the presence of H4 K20 dimethylation (Figure S4). Unexpectedly, although H4 molecules with other degrees of K20 methylation (including H4 molecules that were not K20-methylated) may well exist in DT40 cells, they were not detected in our FAIMS analysis, which suggests that they are far less abundant than H4K20me2. Using a mixture of four synthetic peptides (corresponding to H4K20me0, me1, me2 and me3) that was subjected to propionylation and trypsin digestion, we found that the H4K20me0, me1 and me3 peptides were readily detected using the FAIMS-Orbitrap instrument. Moreover, there was no strong bias in favour of detection of H4K20me2 (data not shown). This demonstrates that the vast majority of H4 molecules are K20-dimethylated in wild-type DT40 cells. Importantly, when normalised to those of a non-modified H4 peptide (not derived from the region containing H4K20), H4K20me2 ion counts were equally abundant in wild-type and Dot1L-deficient cells (Figure S5). Thus, the phenotypes of cells lacking Dot1L cannot be attributed to changes in H4 K20 dimethylation.

### Dot1L deficiency results in proliferation defects in DT40 cells

**Figure 3:**
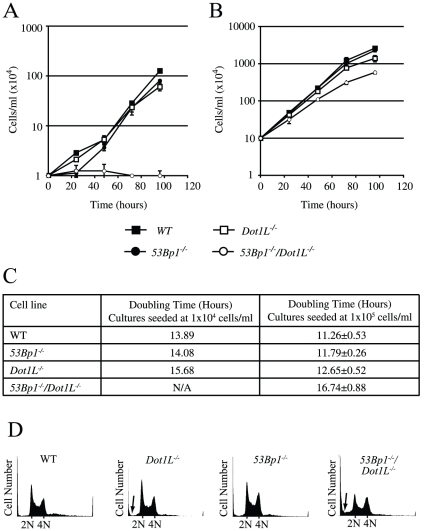
Proliferation and cell cycle distribution of *Dot1L*-deficient cells. A and B. Cell proliferation of cultures seeded at 1×10^4^ cells/ml (A) and 1×10^5^ cells/ml (B). C. Measured doubling times. D. Cell cycle distribution monitored by propidium iodide staining of DNA and flow cytometry. Arrows indicate sub-G1 populations.

Cellular proliferation was examined by monitoring the increase in cell number over time. *Dot1L^−/−^* cells exhibited a mild proliferation defect (Figures 3A and 3B), which was also apparent when doubling times were calculated (Figure 3C). Analysis of cell cycle distribution revealed that disruption of *Dot1L* alone did not greatly affect cell cycle stage distribution in asynchronously proliferating cells (Figure 3D). However a slight increase in the sub-G1 population, which corresponds to dead cells, was observed (Figure 3D). A slight increase in cell death in the absence of functional Dot1L has previously been reported [35].

We have previously reported a mild proliferation defect in 53Bp1 defective DT40 cells [44]. Since Dot1L and H3K79 methylation have been linked to 53Bp1 function in the DNA damage response [20], we asked whether the slow proliferation of *Dot1L^−/−^* cells was due to perturbation of a 53Bp1 pathway. To achieve this, the proliferation rate of the *53Bp1^−/−^/Dot1L^−/−^* cell line, defective in both 53Bp1 and Dot1L, was studied. Interestingly, knockout of *Dot1L* in the *53Bp1^−/−^* cell line led to a severe proliferation defect: *53Bp1^−/−^/Dot1L^−/−^* cultures did not proliferate at all when seeded at a low cell density (Figure 3A), and grew at a slower rate than WT cells or either of the single mutants when seeded at higher cell density (Figure 3B and 3C). A significant increase in the sub-G1 peak was also observed in *53Bp1^−/−^/Dot1L^−/−^* cells (Figure 3D). This suggests that increased levels of cell death occur in the absence of both 53Bp1 and Dot1L, which likely contributes to the pronounced proliferation defect of this cell line.

### 53Bp1 focal recruitment and resistance to ionising radiation in *Dot1L* defective DT40 cells

**Figure 4:**
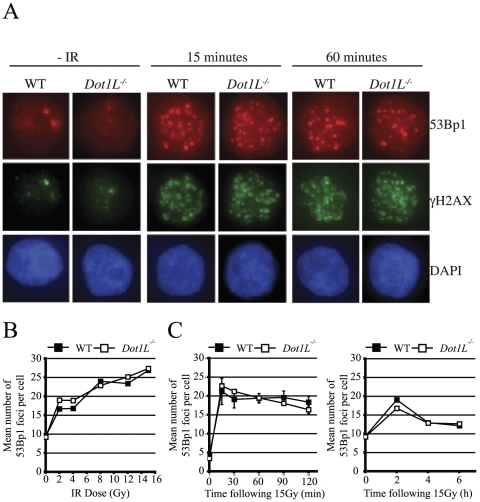
Recruitment of 53Bp1 to nuclear foci following ionising radiation. A. Representative images of 53Bp1 and γH2AX foci obtained by immunofluorescence microscopy in WT and isogenic *Dot1L^−/−^* cells (Cre1 DT40 background). B. Recruitment of 53Bp1 to foci in response to increasing doses of IR was monitored 30 minutes following IR. The number of 53Bp1 foci per cell was counted. 50–100 cells were analysed per data point. C. Kinetics of 53Bp1 foci formation. The number of 53Bp1 foci per cell was counted at the indicated time points following exposure to 15Gy of IR. 50–100 cells were counted per data point. The left panel shows the number of foci per cell from 15 minutes up to 2 hours following IR. The right panel shows the number of 53Bp1 foci per cell from 2 hours up to 6 hours following IR. These quantifications have been obtained from experiments performed on Cre1 DT40 background cell lines.

We next investigated the formation of ionising radiation (IR)-induced foci in cells lacking H3K79 methylation. The formation of foci containing the phosphorylated form of the H2AX histone variant (γ-H2AX), an excellent surrogate marker of DNA double-strand breaks, was not compromised in *Dot1L^−/−^* cells (Figure 4A). In addition, the absence of Dot1L did not enhance the number of γ-H2AX after irradiation of *53Bp1^−/−^* cells (Figure S6).

To determine if H3K79 methylation is required for the recruitment of 53Bp1 to sites of DNA damage, we performed a detailed dose response and time course analysis of 53Bp1 foci formation in DT40-Cre1 and isogenic *Dot1L^−/−^* cells by immunofluorescence microscopy following IR. Despite a slight delay in 53Bp1 foci disappearance at late time points, DT40-Cre1 cells behaved identically to WT cells regarding the kinetics of 53Bp1 accumulation (Figure S7). We observed 53Bp1 focal recruitment in the absence of functional Dot1L at all IR doses and time points tested (Figure 4A). To determine if subtle differences in 53Bp1 recruitment did occur in *Dot1L^−/−^* cells, the mean number of 53Bp1 foci per cell, and the percentage of cells containing 53Bp1 foci were counted. At several doses of IR, the absence of Dot1L and H3K79 methylation did not alter the number of 53Bp1 foci formed per cell (Figure 4B), nor the mean number of cells containing foci (Figure S7E). We also investigated the kinetics of 53Bp1 focal recruitment in the absence of Dot1L. *Dot1L^−/−^* and WT cells were indistinguishable in 53Bp1 focal recruitment and persistence of foci at all time points tested. This was true in terms of the number of 53Bp1 foci per cell (Figures 4 C and D) and the mean number of cells with foci (Figure S7).

**Figure 5:**
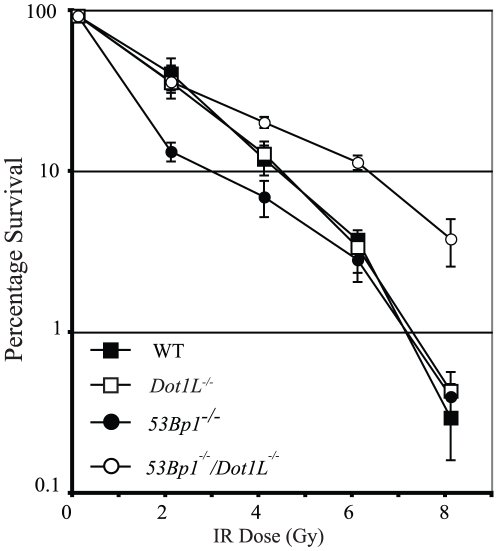
Cell survival following DNA damage caused by ionising radiation. Clonogenic survival of WT, *Dot1L^−/−^, 53Bp1^−/−^*, *and 53Bp1^−/−^/Dot1L^−/−^* cell lines treated with increasing doses of IR. The experiment was carried out three times, error bars show standard deviation.

Sensitivity to DNA damaging agents, which is indicative of DNA repair capability, was also investigated. We have previously reported that 53Bp1 functions in the non-homologous end-joining (NHEJ) DNA double-strand break repair pathway in DT40 cells. 53Bp1-deficient DT40 cells are moderately sensitive to low doses of IR, but resistant to higher doses [44]. Unlike *53Bp1^−/−^* cells, *Dot1L^−/−^* cells were not sensitive to IR, suggesting that these cells are not defective in repair of IR-induced DNA lesions (Figure 5). This is in agreement with results shown in Figures 4C and S7, in which 53Bp1 foci disperse at similar rates in Dot1L-deficient and WT cells. Disappearance of DNA damage foci is thought to correlate with completion of DNA repair. Intriguingly, *53Bp1^−/−^/Dot1L^−/−^* cells exhibited significantly better survival than the *53Bp1^−/−^* single mutant at low doses of IR (Figure 5). At doses of IR greater than 2Gy, *53Bp1^−/−^/Dot1L^−/−^* cells are even more resistant than WT cells (Figure 5). These results indicate that mutation of *Dot1L* reduces IR-induced cell death in the absence of 53Bp1.

### Dot1L affects expression of many genes

**Figure 6:**
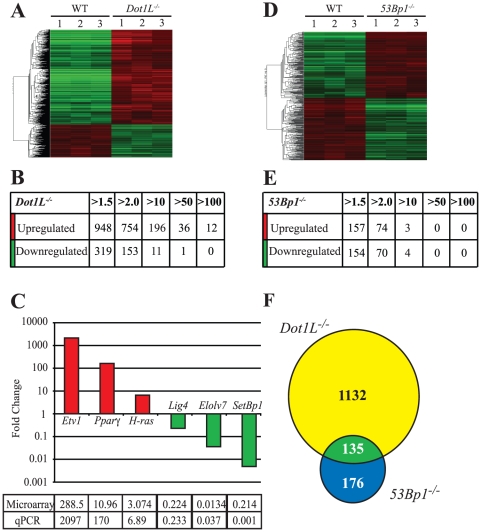
Regulation of gene expression by Dot1L. A. Heat map showing changes in gene expression caused by Dot1L deficiency. In the columns depicting expression data from *Dot1L^−^/^−^* cells, red indicates RNAs that are upregulated in the absence of Dot1L, and green indicates RNAs that are downregulated in the absence of Dot1L. A fold-change cut-off of 1.5 was used. Three technical replicates (numbered 1, 2, and 3) are shown for each cell line. B. Number of RNAs up or downregulated at the indicated fold-change cut-off values in *Dot1L^−/−^* cells. C. Validation of *Dot1L^−/−^* microarray results by RT-qPCR. The graph shows fold change values obtained from qPCR. The table compares fold change values determined using either microarrays or RT-qPCR. Candidate RNAs were chosen to represent the range of fold change up or downregulation observed in the microarray analysis. D. Heat map showing changes in gene expression caused by 53Bp1 deficiency. A fold-change cut-off of 1.5 was used. Three technical replicates (numbered 1, 2, and 3) are shown for each cell line. E. Number of genes up or downregulated at the indicated fold-change cut-off values in *53Bp1^−/−^* cells. F. Overlap of RNAs displaying altered expression levels between WT cells and either *53Bp1^−/−^* or *Dot1L^−/−^*cells.

Because Dot1L is implicated in the regulation of gene expression, we decided to determine the RNA expression profile of *Dot1L^−/−^* cells by microarray analysis. RNA was prepared from WT18 and isogenic *Dot1L^−/−^* DT40 cells and hybridised to Affymetrix Chicken GeneChips. Disruption of *Dot1L* had a dramatic effect on gene expression. The levels of RNAs derived from approximately 1200 genes were significantly altered (more than 1.5-fold) in the absence of functional Dot1L. 74% of these RNAs were upregulated and 26% downregulated in *Dot1L* ^−/−^ cells (Figure 6A and B). The majority of these RNAs displayed a 1.5- to 10-fold differential expression (Figure 6B). We also observed 48 RNAs whose expression levels were elevated by more than 50-fold (Figure 6B). Microarray results were confirmed by reverse transcription and quantitative real-time PCR (RT-qPCR) on candidate RNAs selected on the basis of fold-change upregulation or downregulation (Figure 6C). While the fold change observed by RT-qPCR was in most cases greater than that observed by microarray, the two approaches generated qualitatively similar results (Figure 6C, Table). RT-qPCR analysis of the *Etv1* RNA, which displayed the highest level of upregulation in the absence of Dot1L, showed that this RNA was almost undetectable in WT cells, whereas *Etv1* transcripts were readily detectable in *Dot1L^−/−^*cells. The same was true for another candidate RNA tested, *Ppar*γ. This raises the possibility that these genes may be repressed in WT, and transcriptionally active in *Dot1L^−/−^* cells, although other mechanisms of RNA regulation could also be involved. Conversely, the *Elolv7* and *SetBp1* RNAs were almost undetectable in *Dot1L^−/−^* cells, but were readily detected in WT cells. This likely indicates that these genes are transcriptionally active under normal cellular conditions, but are not expressed in the absence of Dot1L. The *Lig4* and *H-ras* RNAs, which exhibit lower levels of differential expression, were easily detectable in both cell lines. Similar RT-qPCR results were obtained using Dot1L-deficient cells in the DT40-Cre1 background (data not shown).

To further understand the biological relevance of this altered RNA expression profile, a gene ontology (GO) analysis was performed. In agreement with the phenotypes observed in *Dot1L^−/−^* cells, GO groups related to cell proliferation, cell cycle progression and apoptosis were statistically overrepresented in the list of differentially expressed RNAs (Table 1 and Tables S2 and S3). Dot1L is essential for embryonic development and knockout of *Dot1L* is lethal in mice [21], [35]. In this respect it is interesting to note that groups of genes functioning in development and differentiation were also overrepresented in the list of differentially expressed genes.

**Table 1:** Representative sample of gene ontology groups regulated by Dot1L.

GO Term	GO Group	% total genes/GO	% regulated genes/GO	p-value
Upregulated in the absence of Dot1L				
30323	Respiratory tube development	0.137	1.881	2.82×10^−6^
48015	Phosphoinositide-mediated signaling	0.388	2.508	2.82×10^−5^
9653	Morphogenesis	1.924	5.643	4.58×10^−5^
19882	Antigen presentation	0.312	2.194	5.12×10^−5^
30900	Forebrain development	0.144	1.567	7.15×10^−5^
7275	Development	6.288	11.91	1.1×10^−4^
1558	Regulation of cell growth	0.289	1.881	2.79×10^−4^
45639	Positive regulation of myeloid cell differentiation	0.0152	0.627	5.86×10^−4^
1944	Vasculature development	0.312	1.576	0.0029
6979	Response to oxidative stress	0.342	1.576	0.0049
Downregulated in the absence of Dot1L				
50803	Regulation of synapse structure and function	0.0228	1.653	2.50×10^−4^
46689	Response to mercury ion	0.038	1.653	8.25×10^−4^
7264	Small GTPase mediated signal transduction	4.015	10.74	0.0011
6915	Apoptosis	1.719	6.612	0.0011
10035	Response to inorganic substance	0.0608	1.653	0.0023

% total genes/GO indicates the percentage of genes in the GO groups specified in the chicken genome.

% regulated genes/GO indicates the percentage of genes in the specified GO groups that were present in the list of genes whose expression was perturbed in cells lacking Dot1L.

RNA expression profiling was also previously carried out in mouse ES cells in which *Dot1L* was stably knocked down using shRNA [49]. Strikingly, 12% of the RNAs, corresponding to an identified gene, abnormally expressed in *Dot1L* knockdown ES cells were also misregulated in the *Dot1L^−/−^* DT40 cells (see Table S4 for a comparison of the two studies) and similar gene ontology groups were overrepresented. This indicates that the role of Dot1L in the regulation of gene expression is conserved across vertebrate species and distinct cell types. However, we observed that twice as many genes were misregulated in the absence of Dot1L in DT40 cells compared with *Dot1L* knockdown in mouse ES cells, possibly due to residual Dot1L activity resulting from incomplete knockdown (Table S4). Despite this, in both systems, approximately 74% of the differentially expressed RNAs were upregulated in Dot1L-deficient cells (Table S4).

53BP1 interacts with H3K79 methylation [20] and also affects gene expression. The protein binds to the *BRCA1* promoter in human cells and siRNA depletion of *53BP1* results in reduced *BRCA1* mRNA and protein levels [50]. 53BP1 also binds to the tumor suppressor protein p53 and enhances p53-dependent transcription [51], [52]. We performed a microarray analysis on the *53Bp1^−/−^* cell line to determine the impact of 53Bp1 on the global RNA expression profile of DT40 cells. We found that approximately 311 RNAs (compared to 1267 in *Dot1L^−/−^* cells) were differentially expressed between *53Bp1^−/−^*and WT cells, with equal number of RNAs upregulated and downregulated (Figure 6D and E). GO analysis showed that many of these RNAs were involved in the immune response and apoptosis (Tables S5 and S6).

In an attempt to explain the more pronounced cell death and proliferation defect observed in the *53Bp1^−/−^*/*Dot1L^−/−^* cell line (Figure 3), we compared the RNAs whose expression was affected in the *Dot1L^−/−^* and *53Bp1^−/−^* single mutant cells (Figure 6F). 135 of the RNAs inappropriately expressed in *53Bp1^−/−^* cells were also misregulated in *Dot1L^−/−^* cells. This represents 43% of the differentially expressed RNAs in *53Bp1^−/−^* cells and 10% of the RNAs whose expression is altered in *Dot1L^−/−^* cells. The fact that the majority of misregulated RNAs do not overlap is consistent with the additive defects observed in *53Bp1^−/−^*/*Dot1L^−/−^* cells.

In budding yeast, deletion of *DOT1* leads to silencing defects of reporter genes integrated into sub-telomeric heterochromatin [13], [31], [32], [34]. Based on this, we hypothesised that the genes misregulated in the absence of Dot1L may be clustered in chromosomal regions that are either heterochromatic or euchromatic in WT DT40 cells. Although we did not observe any statistically significant clustering of the affected genes (data not shown), we noticed that all but one of the twelve RNAs upregulated by more than 100-fold in cells lacking Dot1L (Figure 6B) were encoded by genes present on macrochromosomes. In chicken, the genome consists of 6 pairs of macrochromosomes, and 30 pairs of microchromosomes [53]. 48% of the genes in chicken are present on macrochromosomes [54]. However, 92% (11 out of 12) of the RNAs that are highly upregulated in the absence of Dot1L are encoded by genes on macrochromosomes. Interestingly, the macrochromsomes are relatively gene-poor compared to the microchromosomes. In addition, genes on macrochromosomes are transcribed at relatively low levels and preferentially located near the periphery of the nucleus [54], [55].

## Discussion

We generated and characterised novel vertebrate cell lines in which the *Dot1L* histone methyltransferase gene has been disrupted. This was achieved by knocking out part of the SAM binding domain of the chicken DT40 gene, which is essential for Dot1L methyltransferase function. We showed that residual levels of *Dot1L* expression are extremely low in these cells. Importantly, the residual *Dot1L* transcripts lack sequences corresponding to exons 5 and 6, which encode the SAM binding domain necessary for enzyme activity. Western blotting and mass spectrometry demonstrated a nearly complete absence of H3K79 methylation in *Dot1L^−/−^* cells: H3K79me2 was undetectable and H3K79me1 was drastically reduced.

*Dot1L* has previously been disrupted in mouse embryonic stem cells using a targeting strategy similar to that employed here [35]. H3K79me1 and H3K79me3 were undetectable. However, a small amount of residual H3K79me2 was reported. The authors postulated that this might be due to contamination from feeder cells necessary to culture ES cells. While it is clear that Dot1L is the main H3K79 methyltransferase in DT40 cells (Figure 2E), we also detect a small amount of residual H3K79me1 in *Dot1L^−/−^*cells. Since our model system does not require the presence of feeder cells, residual H3K79me1 cannot be ascribed to contamination of the *Dot1L^−/−^* culture. An exciting possibility is that an enzyme other than Dot1L is capable of mono-methylating H3K79 on a very small portion of histone H3 molecules. However, we cannot exclude the possibility that the residual H3K79me1 is not physiologically relevant. For example, it may represent a fortuitous off-target product of another methyltransferase with a preference for lysine residues other than H3K79. Alternatively, the low level of residual H3K79me1 in Dot1L-deficient cells may arise through non-enzymatic methylation [56].

Abrogation of Dot1L methyltransferase activity caused defects in DT40 cell proliferation. *Dot1L^−/−^* cells grew at a slower rate than WT cells and flow cytometry analysis suggested increased levels of cell death. It has also been reported that Dot1L is required for normal progression through the cell cycle in mouse ES cells [35], [49], budding yeast [57] and *T. brucei* [58], indicating that some functions of Dot1L are conserved across species and cell types. However, there are also important differences between DT40 and mouse ES cells. First, in contrast to mouse ES cells, H3K79me1 is by far the most prevalent form of H3K79 methylation in DT40 cells. Second, aneuploidy has been reported in Dot1L-deficient mouse ES cells. Most aneuploid ES cells possessed chromosome contents greater than 4N [35], [59]. However, flow cytometry of *Dot1L^−/−^* DT40 cells did not reveal an increased number of cells with a greater than 4N DNA content (Figure 3D). ES cells have less stringent cell cycle checkpoints than differentiated cells [49]. This might explain why DT40 *Dot1L^−/−^* cells do not display any obvious increase in ploidy.

The slight increase in dead cells and slow proliferation of *Dot1L^−/−^* cells might be due to alterations in gene expression. Based on microarray analysis, we found over 1200 RNAs whose expression is perturbed by the absence of Dot1L. 74% of these RNAs were overexpressed in *Dot1L^−/−^* cells, which is consistent with a role for Dot1L in gene silencing and the maintenance of heterochromatin structure. However a significant number of RNAs (26%) were underexpressed in *Dot1L^−/−^*cells. These findings are in agreement with the fact that methylation of H3K79 has been reported to cause both gene activation and repression at different loci [36], [60].

Histone methylation plays an important role in the DNA damage response by contributing to the recruitment of 53BP1 to damaged chromatin. However, much uncertainty did surround the role of H3K79 methylation in the focal recruitment of 53BP1 to sites of DNA damage. The detailed studies that we performed in *Dot1L^−/−^* DT40 cells show that H3K79 methylation is not required for 53Bp1 recruitment to sites of DNA double-strand breaks at the time points and IR doses that we tested. Our results are in agreement with the previously reported role of H4K20me [21], [28], rather than H3K79me, in 53BP1 recruitment to sites of DNA damage. However, it is also possible that, *in vivo*, these two methylation marks function redundantly in 53BP1 recruitment. As stated in the introduction, the function of 53BP1 homologues in budding and fission yeast respectively depends upon H3K79me and H4K20me. This is because only one of the two modifications exists in each yeast model organism. During the course of evolution, vertebrate cells may have retained a function for both types of histone methylation in modulating the recruitment of 53BP1 to damaged chromatin either under specific physiological conditions (*e.g.* different types of DNA lesions) or at distinct loci. This might be consistent with the fact that H3K79me and H4K20me affect chromatin structure in fundamentally different ways [61]. Importantly, the greater abundance of methylated histone H4K20 over H3K79 methylation is consistent with the former modification playing a more prominent role in 53Bp1 recruitment and this is consistent with our observations that Dot1L-deficient cells are not defective in 53Bp1 foci formation. Further work is required to fully elucidate the relative contribution of these two modifications in the DNA damage response.

53BP1 promotes the repair of DNA double-strand breaks by NHEJ [44], [62]. However, DNA double-strand break repair pathways must be functional in the absence of Dot1L since Dot1L-deficient DT40 cells are not sensitive to ionizing radiation. This suggests that H3K79 methylation is not required for the role of 53Bp1 in NHEJ in DT40 cells. However, moderate DNA damage sensitivity has been reported in mouse and human cells lacking DOT1L activity [59]. In contrast, the *53Bp1^−/−^/Dot1L^−/−^*cell line displayed two surprising phenotypes following IR-induced DNA damage. First, at low doses of IR, the *Dot1L^−/−^* mutation rescued the IR sensitivity of *53Bp1^−/−^*cells that are defective in NHEJ (Figure 5). Second, *53Bp1^−/−^/Dot1L^−/−^* cells were even more resistant than WT cells to high doses of IR. One possibility is that the increased IR resistance of cells deficient in both 53Bp1 and Dot1L is a consequence of altered gene expression. Another possibility is that perturbation of heterochromatin structure caused by Dot1L deficiency [35] improves the accessibility of damaged DNA to repair proteins. Although 53BP1 is not a component of the core NHEJ machinery, it is required for a subset of NHEJ repair events [63], [64]. In response to DNA damage, 53BP1 is needed for ATM-dependent phosphorylation of the transcription repressor protein KAP1 [62], [65]. KAP1 directly binds to members of the HP1 protein family and other histone-modifying enzymes that promote heterochromatin formation [63]. Phosphorylation by ATM inactivates KAP1, which leads to a more open heterochromatin structure and facilitates DNA repair [63], [65]. Because Dot1L contributes to heterochromatin structure in mouse ES cells [35], it is conceivable that partial disruption of heterochromatin structure in *Dot1L^−/−^* DT40 cells might alleviate the need for 53Bp1 in Kap1 regulation, thereby facilitating repair of DNA breaks by NHEJ in facultative or constitutive heterochromatic regions (Figure S7). This would explain the rescue of IR resistance in 53Bp1*-*defective cells where Dot1L is also mutated (Figure 5). It has recently been shown that 53BP1 negatively affects HR [66], [67] and mutations that disrupt chromatin structure enhance the efficiency of HR in yeast and mouse cells [68], [69]. In the double mutant, 53Bp1-dependent inhibition of HR will be relieved and absence of Dot1L will result in disrupted heterochromatin that will facilitate NHEJ, independently of the 53Bp1/Kap1 pathway.

In conclusion, we find that Dot1L and H3K79 methylation are required for normal cellular proliferation. Dot1L also strongly influences gene expression. However, Dot1L does not play a major non-redundant role in the focal recruitment of 53Bp1, nor does it function directly in the repair of IR-induced DNA damage. In contrast, in DT40 cells and in the absence of 53Bp1, Dot1L exerts a negative effect on the ability of cells to survive DNA lesions caused by IR.

## Supporting Information

Figure S1 Cross-reaction of antibodies. A. Cross-reaction of antibody recognizing H3K79 monomethylation with unmethylated histone H3. Western blotting was performed on histones prepared from WT and *Dot1L^−/−^* cell lines, as well as recombinant histone H3 as a negative control (upper panel). The western blot was stripped and reprobed with an antibody against total histone H3 to demonstrate equivalent loading (lower panel). B. A mono-methylated histone H3K79 peptide blocks the western blotting signal generated by an antibody supposedly specific for dimethylated H3K79. Western blotting was performed on acid extracted histones prepared from WT and *Dot1L^−/−^* cell lines. Blots were incubated with the antibody against H3K79me2 and different amounts of either H3K79me2 or H3K79me1 peptide as indicated by the peptide/antibody molar ratio. One blot was probed with an antibody against total histone H3 to demonstrate equivalent loading. Similar results were obtained with histones from whole cell extracts (not shown).
(0.87 MB EPS)

Figure S2 Dot1L is conserved in chicken. Alignment of human and chicken Dot1L. Sequences exhibit 73% identity and 92% similarity. Exons are highlighted by alternating blue and black fonts. Amino acids highlighted in red interact with SAM [7], [16]. Amino acids highlighted in green are required for full HMTase activity of hDot1L [16]. The underlined sequence represents the region deleted in the *Dot1L^−/−^* cell line. Note that the sequence of the chicken genome is still missing Exon 1 of *Dot1L*. Therefore, the N-terminal sequence of the chicken Dot1L protein is currently unknown.
(1.45 MB EPS)

Figure S3 FAIMS-MS detection of H4K20 dimethylation. The mass spectrum shows a doubly charged precursor ion with an *m/z* ratio of 272.20 that closely matches the *m/z* ratio predicted for peptide H4 20-(K_me2_ VLR)-23 (where me2 is dimethylation of the lysine ε-amino group). The other peaks correspond to the same doubly charged precursor peptide containing either one (*m/z* 272.70) or two (*m/z* 273.22) naturally occurring ^13^C atoms.
(0.26 MB EPS)

Figure S4 FAIMS-MS/MS detection of H4K20 dimethylation- collision-induced fragmentation spectrum. Collision-induced fragmentation spectrum of the doubly charged precursor ion with *m/z* ratio 272.20 shown in Figure S3. The y1, y2 and y3-ions confirm the sequence H4 21-(VLR)-23 and the b3-ion is consistent with a di-methyl lysine in the sequence H4 20-(K20_me2_VL)-22 (wherein me2 is a dimethylation of the lysine ε-amino group). The abundant *m/z* 249.7 ion corresponds to a neutral loss from the doubly charged precursor peptide of approximately 45 Da. This peak might arise from collision-induced loss of NH(CH3)_2_ at K20, the guanidino group (NH2-CH-NH2) of R23 or the C-terminal COOH.
(0.37 MB EPS)

Figure S5 Comparison of the abundance of H4K20me2 in WT and *Dot1L^md/md^* cells. Extracted ion chromatograms (XIC) from FAIMS-MS experiments showing the abundance of the tryptic peptide 20-(K_me2_VLR)-23 (precursor peptide with m/z 272.202+) in H4 isolated from wild-type (WT) and *Dot1L^md/md^* mutant cells. The indicated ion counts (max intensity) for the H4 20-(K_me2_VLR)-23 peptide in WT and *Dot1L^md/md^* cells are prior to normalisation relative to the ion counts obtained for an H4 peptide that is not modified (DNIQGITKprPAIR). The values obtained after normalisation are indicated above each peak and they are within less than 1% of each other. The experiment was repeated three times with essentially the same result. pr: propionylation of the lysine ε-amino group. CV: compensation voltage. Since no reversed-phase HPLC column was used, elution time refers to the time required for the peptides to be infused and travel through the FAIMS device prior to MS detection.
(0.32 MB EPS)

Figure S6 Formation of γH2AX foci as a function of time following 15Gy IR. Representative images of γH2AX foci obtained by immunofluorescence microscopy before and after exposure to ionizing radiation. Green: γH2AX, blue: DAPI. Comparison of experiments performed with Cre1 DT40 and WT18 cells.
(5.20 MB TIF)

Figure S7 DT 40 WT18 and Cre1 cell lines exhibit identical formation of 53Bp1 foci following IR. A. Representative images of 53Bp1 and γH2AX foci obtained by immunofluorescence microscopy of DT40-WT18 and Cre1 cells. Red: 53Bp1. Green: γH2AX. Blue: DAPI. B and C. Kinetics of 53Bp1 foci formation from 15 minutes to 2 hours following IR (B) and from 2 hours to 6 hours following IR (C) in DT40-WT18, Cre1, and Cre1 *Dot1L^−/−^* cells. The number of 53Bp1 foci per cell was counted at the indicated time points following exposure to 15 Gy IR. 50–100 cells were counted per data point. D and E. Focal recruitment of 53Bp1 at 30 minutes following treatment with increasing doses of IR. Both the mean number of cells with more than 6 foci per cell (D) and the number of 53Bp1 foci per cell (E) were counted. 50–100 cells were analysed per data point.
(2.41 MB EPS)

Figure S8 Schematic representation of the status of heterochromatin and DSB repair in the indicated cell lines. See text for details.
(2.35 MB EPS)

Table S1 Primers used for quantitative real-time PCR validation of microarray results.
(0.07 MB DOCX)

Table S2 GO groups over-represented in *Dot1L*-upregulated genes.
(0.08 MB PDF)

Table S3 GO groups over-represented in *Dot1L*-downregulated genes.
(0.08 MB PDF)

Table S4 Comparison of the number and overlap of genes misregulated in chicken DT40 and mouse ES *Dot1L* deficient cells. ^1^ Up and downregulated. ^2^ Differentially expressed entities from chicken *Dot1L^−/−^* cells (this study) and *Dot1L* knockdown mouse ES cells [49]. ^3^ Number of hits from lists in ^1^ submitted to DAVID Bioinformatics Resources v6.7 with returned gene names [70,71]. ^4^ Number of genes differentially regulated in *Dot1L* knockdown mouse ES cells (from ^3^) for which a homolog is known in chicken. ^5^ Number of genes differentially regulated in both *Dot1L* knockdown mouse ES cells and chicken *Dot1L^−/−^* cells. ^6^ Percentage of the mouse genes misregulated in *Dot1L* knockdown mouse ES cells also misregulated in chicken *Dot1L^−/−^* cells expressed as: number of genes differentially regulated in both *Dot1L* knockdown mouse ES cells and chicken *Dot1L^−/−^* cells^5^ divided by the number of genes differentially regulated in *Dot1L* knockdown mouse ES cells for which a homolog is known in chicken^4^.
(0.05 MB DOCX)

Table S5 GO groups over-represented in *53Bp1*-upregulated genes.
(0.07 MB PDF)

Table S6 GO groups over-represented in *53Bp1*-downregulated genes.
(0.07 MB PDF)
