# Correction: Regulation of the DNA Damage Response and Gene Expression by the Dot1L Histone Methyltransferase and the 53Bp1 Tumour Suppressor

**DOI:** 10.1371/journal.pone.0316233

**Published:** 2024-12-18

**Authors:** Jennifer FitzGerald, Sylvie Moureau, Paul Drogaris, Enda O’Connell, Nebiyu Abshiru, Alain Verreault, Pierre Thibault, Muriel Grenon, Noel F. Lowndes

This notice corrects an error in Fig 2F of this article [1]. The anti-H3 and anti-H3K79me2 *hDot1*L Vector bands represent the same protein on the same western blot, but probed with either an antibody that recognizes the unmodified protein, or a post-translational modification of the same protein. Due to the similarity between the blots, the incorrect results were selected during the preparation of the anti-H3K79me2 *hDot1*L Vector panel.

**Fig 2:**
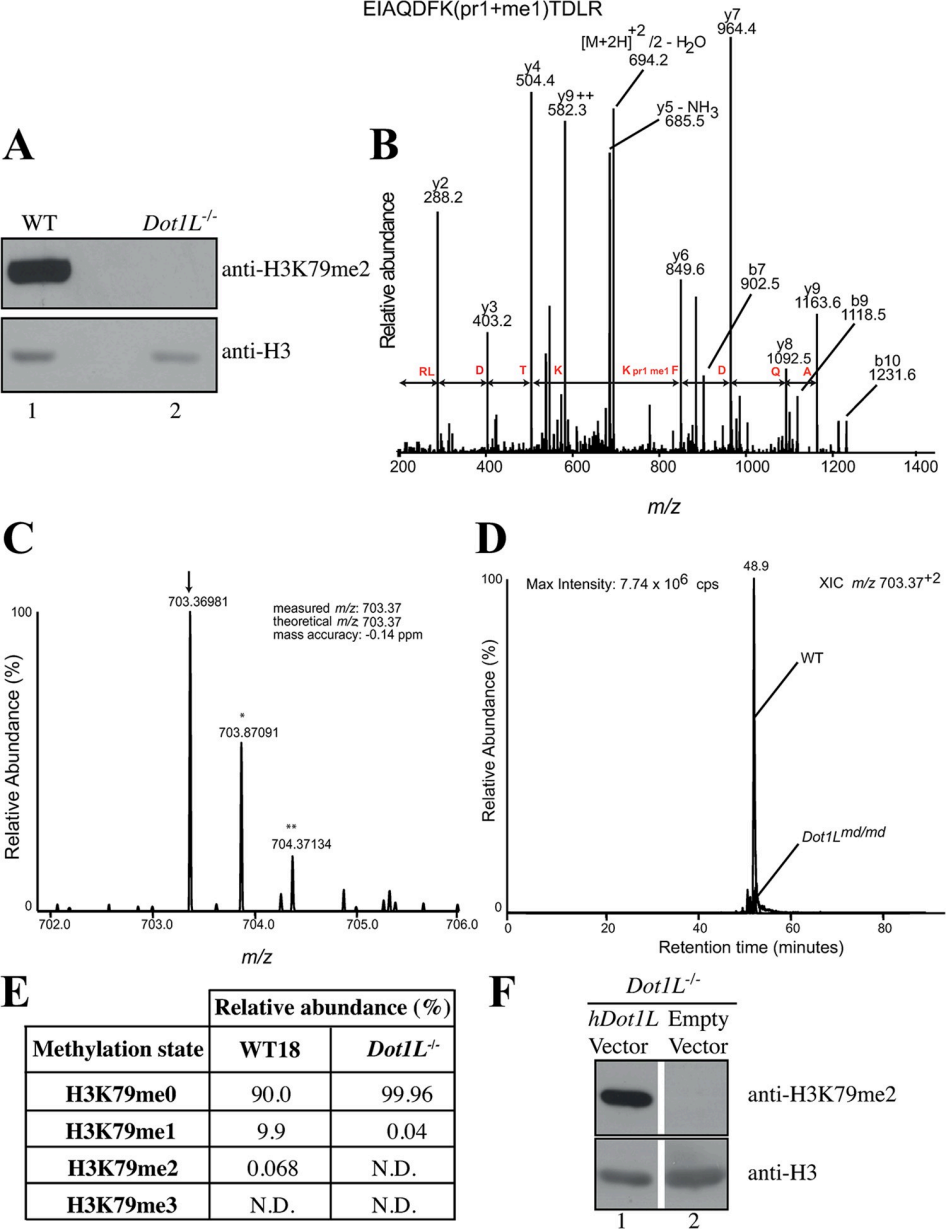
Histone H3 Lysine 79 is not methylated in *Dot1L*^*−/−*^ cells. A. Western blot showing the loss of H3K79 dimethylation in *Dot1L*^*−/−*^ cells. The membrane was stripped and reprobed with an antibody against total H3 to demonstrate equivalent loading. B. Collision-induced fragmentation spectrum of precursor ion *m/z* 706.37^2+^ (shown in C). The spectrum shows a nearly complete y-ion series. The mass difference between the y4 and y6-ion confirms the presence of a single propionyl and methyl group on H3K79 (pr1+me1). C. Doubly charged precursor ion with the measured *m/z* ratio expected for peptide EIAQDFK_79(pr1+me1)_TDLR. The monoisotopic peak containing only ^12^C atoms (arrow) and those containing a single ^13^C (*) or two naturally occurring ^13^C atoms (**) are shown. D. Extracted ion chromatograms (XIC) showing the abundance of the doubly charged precursor peptide containing H3K79me1 (pr1+me1) in WT and *Dot1L*^*−/−*^ cells. E. Relative abundance of the various H3K79 methylation states determined by MRM mass spectrometry in total histones isolated from WT and *Dot1L*^*−/−*^ cells. ND: not detected. F. Western blot showing that the absence of H3K79me2 in *Dot1L*^*−/−*^ cells can be complemented by transient transfection of plasmid containing the human *Dot1L* cDNA.

Fig 2F has been updated to present the correct results. The original blots underlying the Fig 2F results are provided in the S1 File available with this notice.

The corresponding author stated that the remaining data underlying the published results would have been available to *PLOS ONE* in 2014 when a corrected Fig 2F was first provided to the journal. However, the underlying data are now no longer available. PLOS sincerely regrets that this case was not resolved much sooner after the prior correspondence.

The authors apologize for the error in the published article.

## Supporting information

S1 File Raw image data underlying Fig 2F. (DOCX)
