# Supplementary figures and images for: Regulation of the DNA Damage Response and Gene Expression by the Dot1L Histone Methyltransferase and the 53Bp1 Tumour Suppressor
Source: PLoS One. 2011 Feb 24;6(2):e14714. doi: 10.1371/journal.pone.0014714 (PMC3044716; doi:10.1371/journal.pone.0014714)

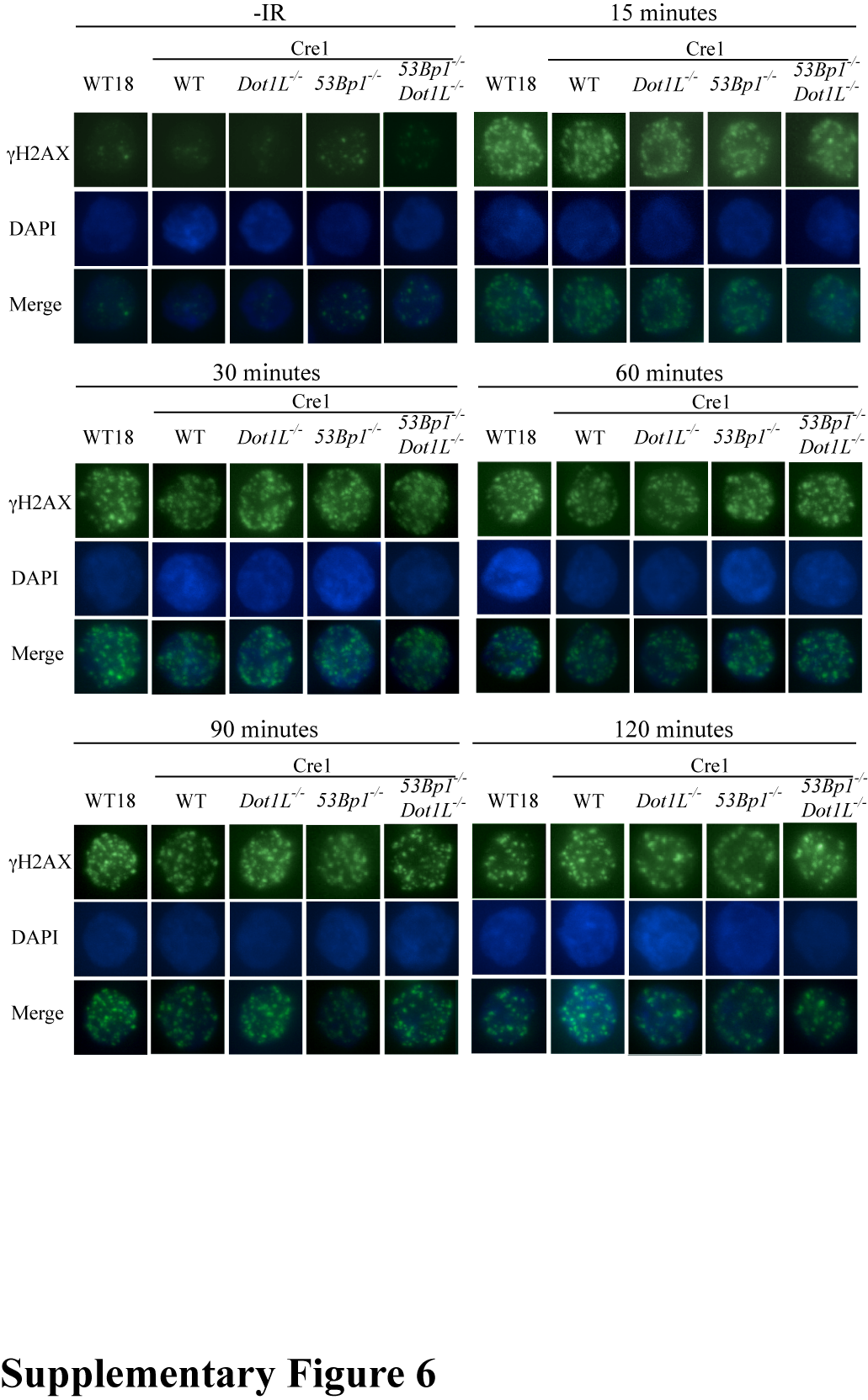

Supplement: Figure S6 — Formation of γH2AX foci as a function of time following 15Gy IR. Representative images of γH2AX foci obtained by immunofluorescence microscopy before and after exposure to ionizing radiation. Green: γH2AX, blue: DAPI. Comparison of experiments performed with Cre1 DT40 and WT18 cells. (5.20 MB TIF) [file pone.0014714.s006.tif]
