# Supplementary material for: Regulation of the DNA Damage Response and Gene Expression by the Dot1L Histone Methyltransferase and the 53Bp1 Tumour Suppressor
Source: PLoS One. 2011 Feb 24;6(2):e14714. doi: 10.1371/journal.pone.0014714 (PMC3044716; doi:10.1371/journal.pone.0014714)
Supplement: Table S1 — Primers used for quantitative real-time PCR validation of microarray results. (0.07 MB DOCX) [file pone.0014714.s009.docx]

**Supplementary Table S1.**

| Gene | Forward/reverse | Primers |
| --- | --- | --- |
| *Dot1L* | Forward | AACAACTACGAGCCCTTCTC |
|  | Reverse | CACCACCTGACCCACACC |
| *Etv1* | Forward | CTCCTCTGATGATTAAACAGGAAC |
|  | Reverse | CTTCTTGCCTCATGTAAATGGAATG |
| *Pparγ* | Forward | ATAAAGTCCTTCCCTCTGACC |
|  | Reverse | TTGATCTGATCTTCTCCCATCC |
| *Lig4* | Forward | GTGGCAGTGTGGTACAGAAC |
|  | Reverse | TCAGCTCCTACAATGACACAG |
| *H-ras* | Forward | TTGCCATTAACAACACCAAGTCC |
|  | Reverse | ACATTTATTTCCCACCAGCACC |
| *Elolv7* | Forward | CTGCATGTCTTTCATCATTCCATC |
|  | Reverse | AGCATGAAACGTTCCTAAGCC |
| *SetBP1* | Forward | GATCCAGACGACCAAGAGAG |
|  | Reverse | GCTTGCTGACCAGTCAAGATG |
| *β-actin* | Forward | TCATCACCATTGGCAATGAGAG |
|  | Reverse | CAGGACTCCATACCCAAGAAAG |
| *GAPDH* | Forward | GCAGATGCAGGTGCTGAG |
|  | Reverse | GGAGCTGAGATGATAACACGC |
