# Supplementary material for: Regulation of the DNA Damage Response and Gene Expression by the Dot1L Histone Methyltransferase and the 53Bp1 Tumour Suppressor
Source: PLoS One. 2011 Feb 24;6(2):e14714. doi: 10.1371/journal.pone.0014714 (PMC3044716; doi:10.1371/journal.pone.0014714)
Supplement: Table S2 — GO groups over-represented in Dot1L-upregulated genes. (0.08 MB PDF) [file pone.0014714.s010.pdf]

Supplementary Table S2: GO groups over-represented in *Dot1L*-upregulated genes

Biological Process Category	Genes in Category	% of Genes in Category	Genes in List in Category	% of Genes in List in Category	p-Value
GO:30323: respiratory tube development	18	0.137	6	1.881	2.82E-06
GO:30324: lung development	18	0.137	6	1.881	2.82E-06
GO:35295: tube development	37	0.281	7	2.194	2.55E-05
GO:48015: phosphoinositide-mediated signaling	51	0.388	8	2.508	2.82E-05
GO:9653: morphogenesis	253	1.924	18	5.643	4.58E-05
GO:19882: antigen presentation	41	0.312	7	2.194	5.12E-05
GO:30900: forebrain development	19	0.144	5	1.567	7.15E-05
GO:30902: hindbrain development	11	0.0836	4	1.254	9.80E-05
GO:7275: development	827	6.288	38	11.91	0.000108
GO:19932: second-messenger-mediated signaling	66	0.502	8	2.508	0.000185
GO:1558: regulation of cell growth	38	0.289	6	1.881	0.000279
GO:48513: organ development	223	1.696	15	4.702	0.000359
GO:45648: positive regulation of erythrocyte differentiation	2	0.0152	2	0.627	0.000587
GO:45639: positive regulation of myeloid cell differentiation	2	0.0152	2	0.627	0.000587
GO:7200: G-protein signaling, coupled to IP3 second messenger (phospholipase C activating	29	0.221	5	1.567	0.000599
GO:7205: protein kinase C activation	29	0.221	5	1.567	0.000599
GO:8361: regulation of cell size	47	0.357	6	1.881	0.000903
GO:16049: cell growth	47	0.357	6	1.881	0.000903
GO:40008: regulation of growth	51	0.388	6	1.881	0.0014
GO:8285: negative regulation of cell proliferation	35	0.266	5	1.567	0.00145
GO:9887: organ morphogenesis	135	1.027	10	3.135	0.00166
GO:7165: signal transduction	2599	19.76	84	26.33	0.00237
GO:1944: vasculature development	41	0.312	5	1.567	0.00298
GO:1568: blood vessel development	41	0.312	5	1.567	0.00298
GO:7420: brain development	41	0.312	5	1.567	0.00298
GO:42127: regulation of cell proliferation	79	0.601	7	2.194	0.00301
GO:7154: cell communication	3265	24.83	101	31.66	0.00314
GO:902: cellular morphogenesis	124	0.943	9	2.821	0.00321
GO:8283: cell proliferation	124	0.943	9	2.821	0.00321
GO:45646: regulation of erythrocyte differentiation	4	0.0304	2	0.627	0.00341
GO:45637: regulation of myeloid cell differentiation	4	0.0304	2	0.627	0.00341
GO:6111: regulation of gluconeogenesis	4	0.0304	2	0.627	0.00341
GO:45722: positive regulation of gluconeogenesis	4	0.0304	2	0.627	0.00341
GO:42541: hemoglobin biosynthesis	4	0.0304	2	0.627	0.00341
GO:42136: neurotransmitter biosynthesis	4	0.0304	2	0.627	0.00341
GO:20027: hemoglobin metabolism	4	0.0304	2	0.627	0.00341
GO:82: G1/S transition of mitotic cell cycle	4	0.0304	2	0.627	0.00341
GO:6979: response to oxidative stress	45	0.342	5	1.567	0.00449
GO:6800: oxygen and reactive oxygen species metabolism	49	0.373	5	1.567	0.00648
GO:7417: central nervous system development	50	0.38	5	1.567	0.00706
GO:6817: phosphate transport	168	1.277	10	3.135	0.0079
GO:48514: blood vessel morphogenesis	34	0.259	4	1.254	0.00888
GO:6334: nucleosome assembly	74	0.563	6	1.881	0.00904
GO:16311: dephosphorylation	227	1.726	12	3.762	0.0095
GO:7498: mesoderm development	7	0.0532	2	0.627	0.0114
GO:43255: regulation of carbohydrate biosynthesis	7	0.0532	2	0.627	0.0114
GO:6109: regulation of carbohydrate metabolism	7	0.0532	2	0.627	0.0114
GO:45913: positive regulation of carbohydrate metabolism	7	0.0532	2	0.627	0.0114
GO:46834: lipid phosphorylation	21	0.16	3	0.94	0.0136
GO:46854: phosphoinositide phosphorylation	21	0.16	3	0.94	0.0136
GO:30218: erythrocyte differentiation	8	0.0608	2	0.627	0.0149
GO:6470: protein amino acid dephosphorylation	189	1.437	10	3.135	0.017
GO:30384: phosphoinositide metabolism	42	0.319	4	1.254	0.0184
GO:30308: negative regulation of cell growth	9	0.0684	2	0.627	0.0189
GO:45792: negative regulation of cell size	9	0.0684	2	0.627	0.0189
GO:31497: chromatin assembly	88	0.669	6	1.881	0.02
GO:30258: lipid modification	65	0.494	5	1.567	0.0205
GO:6265: DNA topological change	25	0.19	3	0.94	0.0219
GO:50678: regulation of epithelial cell proliferation	10	0.076	2	0.627	0.0232
GO:50673: epithelial cell proliferation	10	0.076	2	0.627	0.0232
GO:45926: negative regulation of growth	10	0.076	2	0.627	0.0232
GO:42637: catagen	1	0.0076	1	0.313	0.0243
GO:30307: positive regulation of cell growth	1	0.0076	1	0.313	0.0243
GO:45793: positive regulation of cell size	1	0.0076	1	0.313	0.0243
GO:10002: cardioblast differentiation	1	0.0076	1	0.313	0.0243
GO:48286: alveolus development	1	0.0076	1	0.313	0.0243
GO:6587: serotonin biosynthesis from tryptophan	1	0.0076	1	0.313	0.0243
GO:42416: dopamine biosynthesis	1	0.0076	1	0.313	0.0243
GO:15012: heparan sulfate proteoglycan biosynthesis	1	0.0076	1	0.313	0.0243
GO:30201: heparan sulfate proteoglycan metabolism	1	0.0076	1	0.313	0.0243
GO:45823: positive regulation of heart contraction rate	1	0.0076	1	0.313	0.0243
GO:46902: regulation of mitochondrial membrane permeability	1	0.0076	1	0.313	0.0243
GO:17145: stem cell division	1	0.0076	1	0.313	0.0243
GO:48103: somatic stem cell division	1	0.0076	1	0.313	0.0243
GO:7265: Ras protein signal transduction	1	0.0076	1	0.313	0.0243
GO:6650: glycerophospholipid metabolism	46	0.35	4	1.254	0.0249
GO:7186: G-protein coupled receptor protein signaling pathway	853	6.486	30	9.404	0.0258
GO:30097: hemopoiesis	27	0.205	3	0.94	0.0269
GO:7166: cell surface receptor linked signal transduction	1140	8.669	38	11.91	0.0276
GO:30099: myeloid cell differentiation	11	0.0836	2	0.627	0.0279

GO:40007: growth	95	0.722	6	1.881	0.0279
GO:6461: protein complex assembly	123	0.935	7	2.194	0.0302
GO:9566: fertilization	12	0.0912	2	0.627	0.033
GO:7338: fertilization (sensu Metazoa)	12	0.0912	2	0.627	0.033
GO:30035: microspike biogenesis	12	0.0912	2	0.627	0.033
GO:42133: neurotransmitter metabolism	12	0.0912	2	0.627	0.033
GO:7399: nervous system development	155	1.179	8	2.508	0.0352
GO:1525: angiogenesis	30	0.228	3	0.94	0.0354
GO:30036: actin cytoskeleton organization and biogenesis	107	0.814	6	1.881	0.0458
GO:48731: system development	164	1.247	8	2.508	0.0466
GO:7342: fusion of sperm to egg plasma membrane	2	0.0152	1	0.313	0.0479
GO:42428: serotonin metabolism	2	0.0152	1	0.313	0.0479
GO:42427: serotonin biosynthesis	2	0.0152	1	0.313	0.0479
GO:30166: proteoglycan biosynthesis	2	0.0152	1	0.313	0.0479
GO:1836: release of cytochrome c from mitochondria	2	0.0152	1	0.313	0.0479
GO:8016: regulation of heart contraction rate	2	0.0152	1	0.313	0.0479
GO:30595: immune cell chemotaxis	2	0.0152	1	0.313	0.0479
GO:30593: neutrophil chemotaxis	2	0.0152	1	0.313	0.0479
GO:45026: plasma membrane fusion	2	0.0152	1	0.313	0.0479
GO:48041: focal adhesion formation	2	0.0152	1	0.313	0.0479
GO:46626: regulation of insulin receptor signaling pathway	2	0.0152	1	0.313	0.0479
GO:7212: dopamine receptor signaling pathway	2	0.0152	1	0.313	0.0479
GO:50793: regulation of development	82	0.624	5	1.567	0.0489

**Cellular Component Category**	**Genes in Category**	**% of Genes in Category**	**Genes in List in Category**	**% of Genes in List in Category**	**p-Value**
GO:42613: MHC class II protein complex	20	0.172	6	2.027	7.36E-06
GO:42611: MHC protein complex	39	0.335	6	2.027	0.000413
GO:1772: immunological synapse	40	0.344	6	2.027	0.000475
GO:48180: activin complex	2	0.0172	2	0.676	0.000644
GO:43509: activin A complex	2	0.0172	2	0.676	0.000644
GO:31234: extrinsic to internal side of plasma membrane	2	0.0172	2	0.676	0.000644
GO:30427: site of polarized growth	11	0.0945	3	1.014	0.00231
GO:30426: growth cone	11	0.0945	3	1.014	0.00231
GO:5581: collagen	54	0.464	6	2.027	0.00238
GO:5576: extracellular region	1029	8.837	41	13.85	0.00252
GO:43511: inhibin complex	4	0.0344	2	0.676	0.00374
GO:43512: inhibin A complex	4	0.0344	2	0.676	0.00374
GO:9898: internal side of plasma membrane	5	0.0429	2	0.676	0.00612
GO:786: nucleosome	69	0.593	6	2.027	0.00804
GO:30863: cortical cytoskeleton	18	0.155	3	1.014	0.01
GO:31012: extracellular matrix	450	3.865	20	6.757	0.011
GO:5912: adherens junction	19	0.163	3	1.014	0.0117
GO:5578: extracellular matrix (sensu Metazoa)	435	3.736	19	6.419	0.0154
GO:5615: extracellular space	86	0.739	6	2.027	0.0221
GO:30126: COPI vesicle coat	12	0.103	2	0.676	0.0359
GO:30137: COPI-coated vesicle	12	0.103	2	0.676	0.0359
GO:5942: phosphoinositide 3-kinase complex	29	0.249	3	1.014	0.0365
GO:30055: cell-matrix junction	14	0.12	2	0.676	0.0479
GO:5924: cell-substrate adherens junction	14	0.12	2	0.676	0.0479
GO:5925: focal adhesion	14	0.12	2	0.676	4.79E-02

**Molecular Function Category**	**Genes in Category**	**% of Genes in Category**	**Genes in List in Category**	**% of Genes in List in Category**	**p-Value**
GO:4364: glutathione transferase activity	13	0.0748	6	1.405	3.15E-07
GO:16765: transferase activity, transferring alkyl or aryl (other than methyl) groups	40	0.23	7	1.639	4.77E-05
GO:46934: phosphatidylinositol-4,5-bisphosphate 3-kinase activity	7	0.0403	3	0.703	0.000479
GO:4143: diacylglycerol kinase activity	28	0.161	5	1.171	0.00054
GO:48184: follistatin binding	2	0.0115	2	0.468	0.000603
GO:16209: antioxidant activity	50	0.288	6	1.405	0.00135
GO:4465: lipoprotein lipase activity	3	0.0173	2	0.468	0.00178
GO:4926: non-G-protein coupled 7TM receptor activity	25	0.144	4	0.937	0.00302
GO:15459: potassium channel regulator activity	4	0.023	2	0.468	0.0035
GO:16684: oxidoreductase activity, acting on peroxide as acceptor	43	0.247	5	1.171	0.00391
GO:4601: peroxidase activity	43	0.247	5	1.171	0.00391
GO:8201: heparin binding	27	0.155	4	0.937	0.00403
GO:8092: cytoskeletal protein binding	397	2.285	19	4.45	0.00456
GO:5539: glycosaminoglycan binding	47	0.271	5	1.171	0.00575
GO:1727: lipid kinase activity	31	0.178	4	0.937	0.00669
GO:5201: extracellular matrix structural constituent	71	0.409	6	1.405	0.00794
GO:15457: auxiliary transport protein activity	6	0.0345	2	0.468	0.00847
GO:16247: channel regulator activity	6	0.0345	2	0.468	0.00847
GO:1871: pattern binding	54	0.311	5	1.171	0.0103
GO:30247: polysaccharide binding	54	0.311	5	1.171	0.0103
GO:35004: phosphoinositide 3-kinase activity	19	0.109	3	0.703	0.0107
GO:16303: phosphatidylinositol 3-kinase activity	19	0.109	3	0.703	0.0107
GO:5520: insulin-like growth factor binding	20	0.115	3	0.703	0.0123
GO:5515: protein binding	4406	25.36	129	30.21	0.0124
GO:16791: phosphoric monoester hydrolase activity	352	2.026	16	3.747	0.0139
GO:3779: actin binding	297	1.709	14	3.279	0.0155
GO:4871: signal transducer activity	2242	12.9	70	16.39	0.0201
GO:4428: inositol or phosphatidylinositol kinase activity	43	0.247	4	0.937	0.0209
GO:4033: aldo-keto reductase activity	1	0.00576	1	0.234	0.0246

GO:8106: alcohol dehydrogenase (NADP+) activity	1	0.00576	1	0.234	0.0246
GO:4447: iodide peroxidase activity	1	0.00576	1	0.234	0.0246
GO:16308: 1-phosphatidylinositol-4-phosphate 5-kinase activity	1	0.00576	1	0.234	0.0246
GO:3785: actin monomer binding	1	0.00576	1	0.234	0.0246
GO:51370: ZASP binding	1	0.00576	1	0.234	0.0246
GO:51373: FATZ binding	1	0.00576	1	0.234	0.0246
GO:51374: FATZ 1 binding	1	0.00576	1	0.234	2.46E-02
GO:31267: small GTPase binding	46	0.265	4	0.937	0.0261
GO:8083: growth factor activity	118	0.679	7	1.639	0.0265
GO:4197: cysteine-type endopeptidase activity	123	0.708	7	1.639	0.0323
GO:5102: receptor binding	422	2.429	17	3.981	0.0324
GO:15929: hexosaminidase activity	12	0.0691	2	0.468	0.0338
GO:42578: phosphoric ester hydrolase activity	460	2.648	18	4.215	0.0361
GO:46906: tetrapyrrole binding	183	1.053	9	2.108	0.0373
GO:20037: heme binding	183	1.053	9	2.108	0.0373
GO:4721: phosphoprotein phosphatase activity	242	1.393	11	2.576	0.0374
GO:8158: hedgehog receptor activity	14	0.0806	2	0.468	0.0451
GO:4510: tryptophan 5-monooxygenase activity	2	0.0115	1	0.234	0.0486
GO:3830: beta-1,4-mannosylglycoprotein 4-beta-N-acetylglucosaminyltransferase activity	2	0.0115	1	0.234	0.0486
GO:4478: methionine adenosyltransferase activity	2	0.0115	1	0.234	0.0486
GO:4342: glucosamine-6-phosphate deaminase activity	2	0.0115	1	0.234	0.0486
GO:1540: beta-amyloid binding	2	0.0115	1	0.234	0.0486
