# Supplementary material for: Regulation of the DNA Damage Response and Gene Expression by the Dot1L Histone Methyltransferase and the 53Bp1 Tumour Suppressor
Source: PLoS One. 2011 Feb 24;6(2):e14714. doi: 10.1371/journal.pone.0014714 (PMC3044716; doi:10.1371/journal.pone.0014714)
Supplement: Table S3 — GO groups over-represented in Dot1L-downregulated genes. (0.08 MB PDF) [file pone.0014714.s011.pdf]

**Supplementary Table S3: GO groups over-represented in *Dot1L*-downregulated genes**

| **Biological Process Category** | **Genes in Category** | **% of Genes in Category** | **Genes in List in Category** | **% of Genes in List in Category** | **p-Value** |
|---|---|---|---|---|---|
| GO:50803: regulation of synapse structure and function | 3 | 0.0228 | 2 | 1.653 | 2.50E-04 |
| GO:50804: regulation of synaptic transmission | 3 | 0.0228 | 2 | 1.653 | 0.00025 |
| GO:50805: negative regulation of synaptic transmission | 3 | 0.0228 | 2 | 1.653 | 0.00025 |
| GO:15694: mercury ion transport | 5 | 0.038 | 2 | 1.653 | 0.00083 |
| GO:46689: response to mercury ion | 5 | 0.038 | 2 | 1.653 | 0.00083 |
| GO:50787: detoxification of mercury ion | 5 | 0.038 | 2 | 1.653 | 0.00083 |
| GO:7264: small GTPase mediated signal transduction | 528 | 4.015 | 13 | 10.74 | 0.00113 |
| GO:6915: apoptosis | 226 | 1.719 | 8 | 6.612 | 0.00114 |
| GO:12501: programmed cell death | 227 | 1.726 | 8 | 6.612 | 0.00118 |
| GO:16265: death | 244 | 1.855 | 8 | 6.612 | 0.00186 |
| GO:8219: cell death | 244 | 1.855 | 8 | 6.612 | 0.00186 |
| GO:10035: response to inorganic substance | 8 | 0.0608 | 2 | 1.653 | 0.00227 |
| GO:10038: response to metal ion | 8 | 0.0608 | 2 | 1.653 | 0.00227 |
| GO:7165: signal transduction | 2599 | 19.76 | 37 | 30.58 | 0.00293 |
| GO:42981: regulation of apoptosis | 157 | 1.194 | 6 | 4.959 | 0.00327 |
| GO:43067: regulation of programmed cell death | 158 | 1.201 | 6 | 4.959 | 0.00338 |
| GO:7242: intracellular signaling cascade | 990 | 7.528 | 18 | 14.88 | 0.00404 |
| GO:6825: copper ion transport | 12 | 0.0912 | 2 | 1.653 | 0.00522 |
| GO:19827: stem cell maintenance | 1 | 0.0076 | 1 | 0.826 | 0.0092 |
| GO:35019: somatic stem cell maintenance | 1 | 0.0076 | 1 | 0.826 | 0.0092 |
| GO:51102: DNA ligation during DNA recombination | 1 | 0.0076 | 1 | 0.826 | 0.0092 |
| GO:6297: nucleotide-excision repair, DNA gap filling | 1 | 0.0076 | 1 | 0.826 | 0.0092 |
| GO:51402: programmed cell death, neurons | 1 | 0.0076 | 1 | 0.826 | 0.0092 |
| GO:48146: positive regulation of fibroblast proliferation | 1 | 0.0076 | 1 | 0.826 | 0.0092 |
| GO:46931: pore complex biogenesis | 1 | 0.0076 | 1 | 0.826 | 0.0092 |
| GO:45879: negative regulation of smoothened signaling pathway | 1 | 0.0076 | 1 | 0.826 | 0.0092 |
| GO:50850: positive regulation of calcium-mediated signaling | 1 | 0.0076 | 1 | 0.826 | 0.0092 |
| GO:10165: response to X-ray | 1 | 0.0076 | 1 | 0.826 | 0.0092 |
| GO:9314: response to radiation | 19 | 0.144 | 2 | 1.653 | 0.013 |
| GO:51056: regulation of small GTPase mediated signal transduction | 286 | 2.175 | 7 | 5.785 | 0.0165 |
| GO:45058: T cell selection | 2 | 0.0152 | 1 | 0.826 | 0.0183 |
| GO:45061: thymic T cell selection | 2 | 0.0152 | 1 | 0.826 | 0.0183 |
| GO:45060: negative thymic T cell selection | 2 | 0.0152 | 1 | 0.826 | 0.0183 |
| GO:19673: GDP-mannose metabolism | 2 | 0.0152 | 1 | 0.826 | 0.0183 |
| GO:6266: DNA ligation | 2 | 0.0152 | 1 | 0.826 | 0.0183 |
| GO:51103: DNA ligation during DNA repair | 2 | 0.0152 | 1 | 0.826 | 0.0183 |
| GO:12: single strand break repair | 2 | 0.0152 | 1 | 0.826 | 0.0183 |
| GO:50848: regulation of calcium-mediated signaling | 2 | 0.0152 | 1 | 0.826 | 0.0183 |
| GO:9966: regulation of signal transduction | 367 | 2.791 | 8 | 6.612 | 0.02 |
| GO:6888: ER to Golgi transport | 27 | 0.205 | 2 | 1.653 | 0.0254 |
| GO:6839: mitochondrial transport | 27 | 0.205 | 2 | 1.653 | 0.0254 |
| GO:50789: regulation of biological process | 2486 | 18.9 | 32 | 26.45 | 0.0255 |
| GO:43009: embryonic development (sensu Vertebrata) | 28 | 0.213 | 2 | 1.653 | 0.0272 |
| GO:46649: lymphocyte activation | 28 | 0.213 | 2 | 1.653 | 0.0272 |
| GO:45768: positive regulation of anti-apoptosis | 3 | 0.0228 | 1 | 0.826 | 0.0274 |
| GO:46641: positive regulation of alpha-beta T cell proliferation | 3 | 0.0228 | 1 | 0.826 | 0.0274 |
| GO:46635: positive regulation of alpha-beta T cell activation | 3 | 0.0228 | 1 | 0.826 | 0.0274 |
| GO:46640: regulation of alpha-beta T cell proliferation | 3 | 0.0228 | 1 | 0.826 | 0.0274 |
| GO:51302: regulation of cell division | 3 | 0.0228 | 1 | 0.826 | 0.0274 |
| GO:46633: alpha-beta T cell proliferation | 3 | 0.0228 | 1 | 0.826 | 0.0274 |
| GO:10212: response to ionizing radiation | 3 | 0.0228 | 1 | 0.826 | 0.0274 |
| GO:46634: regulation of alpha-beta T cell activation | 4 | 0.0304 | 1 | 0.826 | 0.0363 |
| GO:48145: regulation of fibroblast proliferation | 4 | 0.0304 | 1 | 0.826 | 0.0363 |
| GO:46631: alpha-beta T cell activation | 4 | 0.0304 | 1 | 0.826 | 0.0363 |
| GO:48144: fibroblast proliferation | 4 | 0.0304 | 1 | 0.826 | 0.0363 |
| GO:8589: regulation of smoothened signaling pathway | 4 | 0.0304 | 1 | 0.826 | 0.0363 |
| GO:50852: T cell receptor signaling pathway | 4 | 0.0304 | 1 | 0.826 | 0.0363 |
| GO:50794: regulation of cellular process | 2365 | 17.98 | 30 | 24.79 | 0.0367 |
| GO:48193: Golgi vesicle transport | 33 | 0.251 | 2 | 1.653 | 0.0368 |
| GO:7154: cell communication | 3265 | 24.83 | 39 | 32.23 | 0.0396 |
| GO:50731: positive regulation of peptidyl-tyrosine phosphorylation | 5 | 0.038 | 1 | 0.826 | 0.0452 |
| GO:50730: regulation of peptidyl-tyrosine phosphorylation | 5 | 0.038 | 1 | 0.826 | 0.0452 |
| GO:45937: positive regulation of phosphate metabolism | 5 | 0.038 | 1 | 0.826 | 0.0452 |
| GO:42327: positive regulation of phosphorylation | 5 | 0.038 | 1 | 0.826 | 0.0452 |
| GO:45767: regulation of anti-apoptosis | 5 | 0.038 | 1 | 0.826 | 0.0452 |
| GO:1775: cell activation | 37 | 0.281 | 2 | 1.653 | 0.0454 |
| GO:45321: immune cell activation | 37 | 0.281 | 2 | 1.653 | 0.0454 |
| GO:9792: embryonic development (sensu Metazoa) | 38 | 0.289 | 2 | 1.653 | 0.0476 |
| GO:8643: carbohydrate transport | 38 | 0.289 | 2 | 1.653 | 0.0476 |
| GO:8284: positive regulation of cell proliferation | 39 | 0.297 | 2 | 1.653 | 0.0499 |
| GO:41: transition metal ion transport | 39 | 0.297 | 2 | 1.653 | 0.0499 |

| **Cellular Component Category** | Genes in Category | **% of Genes in Category** | **Genes in List in Category** | **% of Genes in List in Category** | **p-Value** |
|---|---|---|---|---|---|
| GO:30127: COPII vesicle coat | 14 | 0.12 | 2 | 1.835 | 0.00734 |
| GO:30138: COPII-coated vesicle | 14 | 0.12 | 2 | 1.835 | 0.00734 |
| GO:42101: T cell receptor complex | 1 | 0.00859 | 1 | 0.917 | 0.00936 |
| GO:42105: alpha-beta T cell receptor complex | 1 | 0.00859 | 1 | 0.917 | 0.00936 |
| GO:5834: heterotrimeric G-protein complex | 21 | 0.18 | 2 | 1.835 | 0.0162 |
| GO:19897: extrinsic to plasma membrane | 23 | 0.198 | 2 | 1.835 | 1.93E-02 |
| GO:16020: membrane | 4909 | 42.16 | 56 | 51.38 | 0.032 |
| GO:8091: spectrin | 4 | 0.0344 | 1 | 0.917 | 0.0369 |
| GO:30120: vesicle coat | 34 | 0.292 | 2 | 1.835 | 0.0401 |

| **Molecular Function Category** | **Genes in Category** | **% of Genes in Category** | **Genes in List in Category** | **% of Genes in List in Category** | **p-Value** |
|---|---|---|---|---|---|
| GO:8897: phosphopantetheinyltransferase activity | 2 | 0.0115 | 2 | 1.37 | 7.01E-05 |
| GO:5315: inorganic phosphate transporter activity | 3 | 0.0173 | 2 | 1.37 | 0.00021 |
| GO:4008: copper-exporting ATPase activity | 5 | 0.0288 | 2 | 1.37 | 0.00069 |
| GO:15097: mercury ion transporter activity | 5 | 0.0288 | 2 | 1.37 | 0.00069 |
| GO:30552: 3',5'-cAMP binding | 7 | 0.0403 | 2 | 1.37 | 0.00143 |

| | | | | | |
|---|---|---|---|---|---|
| GO:17124: SH3 domain binding | 7 | 0.0403 | 2 | 1.37 | 0.00143 |
| GO:16712: oxidoreductase activity, acting on paired donors, with incorporation or reduction of molecular oxygen | 32 | 0.184 | 3 | 2.055 | 0.00241 |
| GO:30551: cyclic nucleotide binding | 9 | 0.0518 | 2 | 1.37 | 0.00243 |
| GO:15114: phosphate transporter activity | 11 | 0.0633 | 2 | 1.37 | 0.00367 |
| GO:5375: copper ion transporter activity | 12 | 0.0691 | 2 | 1.37 | 0.00438 |
| GO:16780: phosphotransferase activity, for other substituted phosphate groups | 16 | 0.0921 | 2 | 1.37 | 0.00779 |
| GO:3868: 4-hydroxyphenylpyruvate dioxygenase activity | 1 | 0.00576 | 1 | 0.685 | 0.0084 |
| GO:8432: JUN kinase binding | 1 | 0.00576 | 1 | 0.685 | 0.0084 |
| GO:16705: oxidoreductase activity, acting on paired donors, with incorporation or reduction of molecular oxygen | 96 | 0.553 | 4 | 2.74 | 0.00875 |
| GO:46915: transition metal ion transporter activity | 17 | 0.0978 | 2 | 1.37 | 0.00878 |
| GO:16208: AMP binding | 18 | 0.104 | 2 | 1.37 | 0.00982 |
| GO:30234: enzyme regulator activity | 843 | 4.852 | 14 | 9.589 | 0.0116 |
| GO:4497: monooxygenase activity | 115 | 0.662 | 4 | 2.74 | 0.0161 |
| GO:8446: GDP-mannose 4,6-dehydratase activity | 2 | 0.0115 | 1 | 0.685 | 0.0167 |
| GO:8138: protein tyrosine/serine/threonine phosphatase activity | 66 | 0.38 | 3 | 2.055 | 0.0181 |
| GO:5386: carrier activity | 751 | 4.323 | 12 | 8.219 | 0.0246 |
| GO:5351: sugar porter activity | 32 | 0.184 | 2 | 1.37 | 0.0295 |
| GO:43167: ion binding | 4245 | 24.43 | 46 | 31.51 | 0.0311 |
| GO:3950: NAD+ ADP-ribosyltransferase activity | 33 | 0.19 | 2 | 1.37 | 0.0312 |
| GO:4185: serine carboxypeptidase activity | 4 | 0.023 | 1 | 0.685 | 0.0332 |
| GO:15928: fucosidase activity | 4 | 0.023 | 1 | 0.685 | 0.0332 |
| GO:4560: alpha-L-fucosidase activity | 4 | 0.023 | 1 | 0.685 | 0.0332 |
| GO:51059: NF-kappaB binding | 4 | 0.023 | 1 | 0.685 | 0.0332 |
| GO:19904: protein domain specific binding | 35 | 0.201 | 2 | 1.37 | 0.0348 |
| GO:5085: guanyl-nucleotide exchange factor activity | 289 | 1.663 | 6 | 4.11 | 0.0354 |
| GO:15082: di-, tri-valent inorganic cation transporter activity | 36 | 0.207 | 2 | 1.37 | 0.0367 |
| GO:46872: metal ion binding | 4185 | 24.09 | 45 | 30.82 | 0.0374 |
| GO:5083: small GTPase regulator activity | 460 | 2.648 | 8 | 5.479 | 0.0406 |
| GO:30695: GTPase regulator activity | 545 | 3.137 | 9 | 6.164 | 0.0407 |
| GO:8191: metalloendopeptidase inhibitor activity | 5 | 0.0288 | 1 | 0.685 | 0.0413 |
| GO:19887: protein kinase regulator activity | 40 | 0.23 | 2 | 1.37 | 0.0444 |
| GO:9055: electron carrier activity | 313 | 1.802 | 6 | 4.11 | 0.0489 |
| GO:16715: oxidoreductase activity, acting on paired donors, with incorporation or reduction of molecular oxygen | 6 | 0.0345 | 1 | 0.685 | 0.0494 |
| GO:4500: dopamine beta-monooxygenase activity | 6 | 0.0345 | 1 | 0.685 | 0.0494 |
| GO:8839: dihydrodipicolinate reductase activity | 6 | 0.0345 | 1 | 0.685 | 0.0494 |
| GO:4810: tRNA adenylyltransferase activity | 6 | 0.0345 | 1 | 0.685 | 0.0494 |
| GO:16437: tRNA cytidylyltransferase activity | 6 | 0.0345 | 1 | 0.685 | 0.0494 |
| GO:8929: methylglyoxal synthase activity | 6 | 0.0345 | 1 | 0.685 | 0.0494 |
| GO:5338: nucleotide-sugar transporter activity | 6 | 0.0345 | 1 | 0.685 | 0.0494 |
| GO:19900: kinase binding | 6 | 0.0345 | 1 | 0.685 | 0.0494 |
| GO:19901: protein kinase binding | 6 | 0.0345 | 1 | 0.685 | 0.0494 |
| GO:19210: kinase inhibitor activity | 6 | 0.0345 | 1 | 0.685 | 0.0494 |
| GO:4860: protein kinase inhibitor activity | 6 | 0.0345 | 1 | 0.685 | 0.0494 |
| GO:16538: cyclin-dependent protein kinase regulator activity | 6 | 0.0345 | 1 | 0.685 | 0.0494 |
