# Supplementary material for: Regulation of the DNA Damage Response and Gene Expression by the Dot1L Histone Methyltransferase and the 53Bp1 Tumour Suppressor
Source: PLoS One. 2011 Feb 24;6(2):e14714. doi: 10.1371/journal.pone.0014714 (PMC3044716; doi:10.1371/journal.pone.0014714)
Supplement: Table S4 — Comparison of the number and overlap of genes misregulated in chicken DT40 and mouse ES Dot1L deficient cells. 1 Up and downregulated. 2 Differentially expressed entities from chicken Dot1L−/− cells (this study) and Dot1L knockdown mouse ES cells [49]. 3 Number of hits from lists in 1 submitted to DAVID Bioinformatics Resources v6.7 with returned gene names [70,71]. 4 Number of genes differentially regulated in Dot1L knockdown mouse ES cells (from 3) for which a homolog is known in chicken. 5 Number of genes differentially regulated in both Dot1L knockdown mouse ES cells and chicken Dot1L−/− cells. 6 Percentage of the mouse genes misregulated in Dot1L knockdown mouse ES cells also misregulated in chicken Dot1L−/− cells expressed as: number of genes differentially regulated in both Dot1L knockdown mouse ES cells and chicken Dot1L−/− cells5 divided by the number of genes differentially regulated in Dot1L knockdown mouse ES cells for which a homolog is known in chicken4. (0.05 MB DOCX) [file pone.0014714.s012.docx]

**Supplementary Table S4**

|  | | Number of hits ^2^ | Number of genes^3^ | Misregulated mouse genes with a chicken homologue ^4^ | Number of genes misregulated in both species ^5^ | % ^6^ |
| --- | --- | --- | --- | --- | --- | --- |
| Upregulated | chicken | 948 | 585 | 85 | 12 | 14 |
|  | mouse | 211 | 109 |  |  |  |
| Downregulated | chicken | 319 | 245 | 29 | 2 | 7 |
|  | mouse | 65 | 46 |  |  |  |
| Misregulated^1^ | chicken | 1267 | 830 | 114 | 14 | 12 |
|  | mouse | 276 | 155 |  |  |  |
