# Supplementary material for: Regulation of the DNA Damage Response and Gene Expression by the Dot1L Histone Methyltransferase and the 53Bp1 Tumour Suppressor
Source: PLoS One. 2011 Feb 24;6(2):e14714. doi: 10.1371/journal.pone.0014714 (PMC3044716; doi:10.1371/journal.pone.0014714)
Supplement: Table S5 — GO groups over-represented in 53Bp1-upregulated genes. (0.07 MB PDF) [file pone.0014714.s013.pdf]

Supplementary Table S5: GO groups over-represented in *53Bp1*-upregulated genes

Biological Process Category	Genes in Category	% of Genes in Category	Genes in List in Category	% of Genes in List in Category	p-Value
GO:19882: antigen presentation	41	0.312	4	5.882	5.73E-05
GO:7165: signal transduction	2599	19.76	27	39.71	0.00012
GO:7243: protein kinase cascade	51	0.388	4	5.882	0.00014
GO:50819: negative regulation of coagulation	8	0.0608	2	2.941	0.00072
GO:7229: integrin-mediated signaling pathway	35	0.266	3	4.412	0.00077
GO:7154: cell communication	3265	24.83	29	42.65	0.00095
GO:50818: regulation of coagulation	10	0.076	2	2.941	0.00115
GO:6952: defense response	303	2.304	6	8.824	0.00471
GO:8272: sulfate transport	21	0.16	2	2.941	0.00519
GO:9607: response to biotic stimulus	312	2.372	6	8.824	0.00542
GO:6955: immune response	248	1.886	5	7.353	0.0091
GO:9268: response to pH	2	0.0152	1	1.471	0.0103
GO:10043: response to zinc ion	2	0.0152	1	1.471	0.0103
GO:6787: porphyrin catabolism	4	0.0304	1	1.471	0.0205
GO:6788: heme oxidation	4	0.0304	1	1.471	0.0205
GO:43407: negative regulation of MAPK activity	4	0.0304	1	1.471	0.0205
GO:6812: cation transport	587	4.464	7	10.29	0.0315
GO:51348: negative regulation of transferase activity	7	0.0532	1	1.471	0.0356
GO:6469: negative regulation of protein kinase activity	7	0.0532	1	1.471	0.0356
GO:43405: regulation of MAPK activity	7	0.0532	1	1.471	0.0356
GO:3: reproduction	61	0.464	2	2.941	0.0396
GO:6584: catecholamine metabolism	8	0.0608	1	1.471	0.0406
GO:10035: response to inorganic substance	8	0.0608	1	1.471	0.0406
GO:10038: response to metal ion	8	0.0608	1	1.471	0.0406
GO:30001: metal ion transport	493	3.749	6	8.824	0.0417
GO:9077: histidine family amino acid catabolism	9	0.0684	1	1.471	0.0456
GO:6548: histidine catabolism	9	0.0684	1	1.471	0.0456

Cellular Component Category	Genes in Category	% of Genes in Category	Genes in List in Category	% of Genes in List in Category	p-Value
GO:42613: MHC class II protein complex	20	0.172	3	4.688	0.00017
GO:42611: MHC protein complex	39	0.335	3	4.688	0.00126
GO:8305: integrin complex	39	0.335	3	4.688	0.00126
GO:1772: immunological synapse	40	0.344	3	4.688	0.00135
GO:43235: receptor complex	50	0.429	3	4.688	0.00258
GO:31224: intrinsic to membrane	2937	25.22	25	39.06	0.01
GO:5887: integral to plasma membrane	258	2.216	5	7.812	0.0135
GO:31226: intrinsic to plasma membrane	259	2.224	5	7.812	0.0137
GO:16021: integral to membrane	2872	24.67	24	37.5	0.0151
GO:30286: dynein complex	41	0.352	2	3.125	0.0213
GO:5604: basement membrane	42	0.361	2	3.125	0.0222
GO:16020: membrane	4909	42.16	35	54.69	0.0289
GO:5886: plasma membrane	795	6.828	9	14.06	0.0289

Molecular Function Category	Genes in Category	% of Genes in Category	Genes in List in Category	% of Genes in List in Category	p-Value
GO:30161: calpain inhibitor activity	2	0.0115	2	2.222	2.65E-05
GO:4792: thiosulfate sulfurtransferase activity	5	0.0288	2	2.222	0.00026
GO:4859: phospholipase inhibitor activity	14	0.0806	2	2.222	0.00232
GO:4869: cysteine protease inhibitor activity	15	0.0863	2	2.222	0.00267
GO:5544: calcium-dependent phospholipid binding	18	0.104	2	2.222	0.00385
GO:5543: phospholipid binding	126	0.725	4	4.444	0.00417
GO:16782: transferase activity, transferring sulfur-containing groups	63	0.363	3	3.333	0.00427
GO:287: magnesium ion binding	212	1.22	5	5.556	0.0049
GO:5183: luteinizing hormone-releasing factor activity	1	0.00576	1	1.111	0.00518
GO:31530: gonadotropin-releasing hormone receptor binding	1	0.00576	1	1.111	0.00518
GO:3785: actin monomer binding	1	0.00576	1	1.111	0.00518
GO:16783: sulfurtransferase activity	22	0.127	2	2.222	0.00573
GO:46873: metal ion transporter activity	71	0.409	3	3.333	0.00596
GO:8289: lipid binding	228	1.312	5	5.556	0.00663
GO:42132: fructose-bisphosphatase activity	2	0.0115	1	1.111	0.0103
GO:51428: peptide hormone receptor binding	2	0.0115	1	1.111	0.0103
GO:30171: voltage-gated proton channel activity	2	0.0115	1	1.111	0.0103
GO:42289: MHC class II protein binding	3	0.0173	1	1.111	0.0155
GO:8092: cytoskeletal protein binding	397	2.285	6	6.667	0.0171
GO:4392: heme oxygenase (decyclizing) activity	4	0.023	1	1.111	0.0206
GO:4857: enzyme inhibitor activity	205	1.18	4	4.444	0.0219
GO:16715: oxidoreductase activity, acting on paired donors	6	0.0345	1	1.111	0.0307
GO:4500: dopamine beta-monooxygenase activity	6	0.0345	1	1.111	0.0307
GO:8967: phosphoglycolate phosphatase activity	6	0.0345	1	1.111	0.0307
GO:19203: carbohydrate phosphatase activity	7	0.0403	1	1.111	0.0357
GO:42287: MHC protein binding	7	0.0403	1	1.111	0.0357
GO:16791: phosphoric monoester hydrolase activity	352	2.026	5	5.556	0.0361
