# Supplementary material for: Regulation of the DNA Damage Response and Gene Expression by the Dot1L Histone Methyltransferase and the 53Bp1 Tumour Suppressor
Source: PLoS One. 2011 Feb 24;6(2):e14714. doi: 10.1371/journal.pone.0014714 (PMC3044716; doi:10.1371/journal.pone.0014714)
Supplement: Table S6 — GO groups over-represented in 53Bp1-downregulated genes. (0.07 MB PDF) [file pone.0014714.s014.pdf]

Supplementary Table S6: GO groups over-represented in *53Bp1*-downregulated genes

Biological Process Category	Genes in Category	% of Genes in Category	Genes in List in Category	% of Genes in List in Category	p-Value
GO:15674: di-, tri-valent inorganic cation transport	172	1.308	5	7.463	0.00181
GO:6879: iron ion homeostasis	15	0.114	2	2.985	0.00257
GO:46916: transition metal ion homeostasis	17	0.129	2	2.985	0.00331
GO:6826: iron ion transport	20	0.152	2	2.985	0.00458
GO:30574: collagen catabolism	1	0.0076	1	1.493	0.00509
GO:45879: negative regulation of smoothened signaling pathway	1	0.0076	1	1.493	0.00509
GO:50850: positive regulation of calcium-mediated signaling	1	0.0076	1	1.493	0.00509
GO:9968: negative regulation of signal transduction	29	0.221	2	2.985	0.0095
GO:45058: T cell selection	2	0.0152	1	1.493	0.0102
GO:45061: thymic T cell selection	2	0.0152	1	1.493	0.0102
GO:45060: negative thymic T cell selection	2	0.0152	1	1.493	0.0102
GO:1955: blood vessel maturation	2	0.0152	1	1.493	0.0102
GO:50848: regulation of calcium-mediated signaling	2	0.0152	1	1.493	0.0102
GO:9156: ribonucleoside monophosphate biosynthesis	31	0.236	2	2.985	0.0108
GO:9161: ribonucleoside monophosphate metabolism	31	0.236	2	2.985	0.0108
GO:9124: nucleoside monophosphate biosynthesis	32	0.243	2	2.985	0.0115
GO:9123: nucleoside monophosphate metabolism	32	0.243	2	2.985	0.0115
GO:6875: metal ion homeostasis	33	0.251	2	2.985	0.0122
GO:30005: di-, tri-valent inorganic cation homeostasis	33	0.251	2	2.985	0.0122
GO:30003: cation homeostasis	36	0.274	2	2.985	0.0144
GO:6873: cell ion homeostasis	37	0.281	2	2.985	0.0152
GO:42450: arginine biosynthesis via ornithine	3	0.0228	1	1.493	0.0152
GO:6591: ornithine metabolism	3	0.0228	1	1.493	0.0152
GO:46641: positive regulation of alpha-beta T cell proliferation	3	0.0228	1	1.493	0.0152
GO:46635: positive regulation of alpha-beta T cell activation	3	0.0228	1	1.493	0.0152
GO:46640: regulation of alpha-beta T cell proliferation	3	0.0228	1	1.493	0.0152
GO:46633: alpha-beta T cell proliferation	3	0.0228	1	1.493	0.0152
GO:41: transition metal ion transport	39	0.297	2	2.985	0.0168
GO:19882: antigen presentation	41	0.312	2	2.985	0.0185
GO:6525: arginine metabolism	4	0.0304	1	1.493	0.0202
GO:6526: arginine biosynthesis	4	0.0304	1	1.493	0.0202
GO:46634: regulation of alpha-beta T cell activation	4	0.0304	1	1.493	0.0202
GO:46631: alpha-beta T cell activation	4	0.0304	1	1.493	0.0202
GO:8589: regulation of smoothened signaling pathway	4	0.0304	1	1.493	0.0202
GO:50852: T cell receptor signaling pathway	4	0.0304	1	1.493	0.0202
GO:9116: nucleoside metabolism	45	0.342	2	2.985	0.022
GO:8152: metabolism	7791	59.24	48	71.64	0.0241
GO:50731: positive regulation of peptidyl-tyrosine phosphorylation	5	0.038	1	1.493	0.0252
GO:50730: regulation of peptidyl-tyrosine phosphorylation	5	0.038	1	1.493	0.0252
GO:45937: positive regulation of phosphate metabolism	5	0.038	1	1.493	0.0252
GO:42327: positive regulation of phosphorylation	5	0.038	1	1.493	0.0252
GO:6812: cation transport	587	4.464	7	10.45	0.0293
GO:42102: positive regulation of T cell proliferation	6	0.0456	1	1.493	0.0302
GO:7224: smoothened signaling pathway	6	0.0456	1	1.493	0.0302
GO:50851: antigen receptor-mediated signaling pathway	6	0.0456	1	1.493	0.0302
GO:6816: calcium ion transport	133	1.011	3	4.478	0.0303
GO:42129: regulation of T cell proliferation	7	0.0532	1	1.493	0.0351
GO:42098: T cell proliferation	7	0.0532	1	1.493	0.0351
GO:30001: metal ion transport	493	3.749	6	8.955	0.0392
GO:19794: nonprotein amino acid metabolism	8	0.0608	1	1.493	0.04
GO:6108: malate metabolism	8	0.0608	1	1.493	0.04
GO:50801: ion homeostasis	66	0.502	2	2.985	0.0445
GO:51: urea cycle intermediate metabolism	9	0.0684	1	1.493	0.0449
GO:1934: positive regulation of protein amino acid phosphorylation	9	0.0684	1	1.493	0.0449
GO:45764: positive regulation of amino acid metabolism	9	0.0684	1	1.493	0.0449
GO:50671: positive regulation of lymphocyte proliferation	9	0.0684	1	1.493	0.0449
GO:50870: positive regulation of T cell activation	9	0.0684	1	1.493	0.0449
GO:42981: regulation of apoptosis	157	1.194	3	4.478	0.0459
GO:43067: regulation of programmed cell death	158	1.201	3	4.478	0.0466
GO:6508: proteolysis	937	7.125	9	13.43	0.0472
GO:43010: eye development (sensu Vertebrata)	10	0.076	1	1.493	0.0498
GO:18108: peptidyl-tyrosine phosphorylation	10	0.076	1	1.493	0.0498
GO:18212: peptidyl-tyrosine modification	10	0.076	1	1.493	0.0498
GO:50670: regulation of lymphocyte proliferation	10	0.076	1	1.493	0.0498

Cellular Component Category	Genes in Category	% of Genes in Category	Genes in List in Category	% of Genes in List in Category	p-Value
GO:1772: immunological synapse	40	0.344	3	6.25	0.000584
GO:42101: T cell receptor complex	1	0.00859	1	2.083	0.00412
GO:42105: alpha-beta T cell receptor complex	1	0.00859	1	2.083	0.00412
GO:42611: MHC protein complex	39	0.335	2	4.167	0.0112

Molecular Function Category	Genes in Category	% of Genes in Category	Genes in List in Category	% of Genes in List in Category	p-Value
GO:3824: catalytic activity	6632	38.17	44	58.67	0.000249
GO:4697: protein kinase C activity	8	0.046	2	2.667	0.000506
GO:1565: phorbol ester receptor activity	8	0.046	2	2.667	0.000506
GO:4749: ribose phosphate diphosphokinase activity	12	0.0691	2	2.667	0.00118
GO:8199: ferric iron binding	14	0.0806	2	2.667	0.00162
GO:16778: diphosphotransferase activity	15	0.0863	2	2.667	0.00186
GO:5388: calcium-transporting ATPase activity	19	0.109	2	2.667	0.003
GO:15085: calcium ion transporter activity	19	0.109	2	2.667	0.003
GO:3868: 4-hydroxyphenylpyruvate dioxygenase activity	1	0.00576	1	1.333	0.00432

GO:17147: Wnt-protein binding	1	0.00576	1	1.333	0.00432
GO:8237: metallopeptidase activity	249	1.433	5	6.667	0.00444
GO:4176: ATP-dependent peptidase activity	29	0.167	2	2.667	0.00692
GO:4056: argininosuccinate lyase activity	2	0.0115	1	1.333	0.00862
GO:8233: peptidase activity	833	4.795	9	12	0.00947
GO:15662: ATPase activity, coupled to transmembrane movement of ions	102	0.587	3	4	0.00977
GO:15082: di-, tri-valent inorganic cation transporter activity	36	0.207	2	2.667	0.0105
GO:166: nucleotide binding	3337	19.21	23	30.67	0.0116
GO:16842: amidine-lyase activity	3	0.0173	1	1.333	0.0129
GO:287: magnesium ion binding	212	1.22	4	5.333	0.0133
GO:4175: endopeptidase activity	474	2.728	6	8	0.0165
GO:15928: fucosidase activity	4	0.023	1	1.333	0.0172
GO:4560: alpha-L-fucosidase activity	4	0.023	1	1.333	0.0172
GO:19200: carbohydrate kinase activity	48	0.276	2	2.667	0.0182
GO:17076: purine nucleotide binding	2916	16.78	20	26.67	0.0204
GO:8191: metalloendopeptidase inhibitor activity	5	0.0288	1	1.333	0.0214
GO:42625: ATPase activity, coupled to transmembrane movement of ions	144	0.829	3	4	0.0244
GO:16840: carbon-nitrogen lyase activity	6	0.0345	1	1.333	0.0256
GO:4866: endopeptidase inhibitor activity	151	0.869	3	4	0.0276
GO:43167: ion binding	4245	24.43	26	34.67	0.0299
GO:19829: cation-transporting ATPase activity	63	0.363	2	2.667	0.0303
GO:16491: oxidoreductase activity	878	5.054	8	10.67	0.0351
GO:15405: P-P-bond-hydrolysis-driven transporter activity	167	0.961	3	4	0.0356
GO:30414: protease inhibitor activity	168	0.967	3	4	0.0361
GO:46873: metal ion transporter activity	71	0.409	2	2.667	0.0377
GO:16615: malate dehydrogenase activity	10	0.0576	1	1.333	0.0423
GO:16818: hydrolase activity, acting on acid anhydrides, in phosphorus-containing anhydrides	757	4.357	7	9.333	0.0446
GO:16817: hydrolase activity, acting on acid anhydrides	761	4.38	7	9.333	0.0457
GO:42626: ATPase activity, coupled to transmembrane movement of substances	185	1.065	3	4	0.0459
GO:8252: nucleotidase activity	11	0.0633	1	1.333	0.0465
GO:8253: 5'-nucleotidase activity	11	0.0633	1	1.333	0.0465
GO:43492: ATPase activity, coupled to movement of substances	186	1.071	3	4	0.0465
GO:16820: hydrolase activity, acting on acid anhydrides, catalyzing transmembrane move	189	1.088	3	4	0.0484
