# Supplementary figures and images for: Correction: Regulation of the DNA Damage Response and Gene Expression by the Dot1L Histone Methyltransferase and the 53Bp1 Tumour Suppressor
Source: PLoS One. 2024 Dec 18;19(12):e0316233. doi: 10.1371/journal.pone.0316233 (PMC11654950; doi:10.1371/journal.pone.0316233)

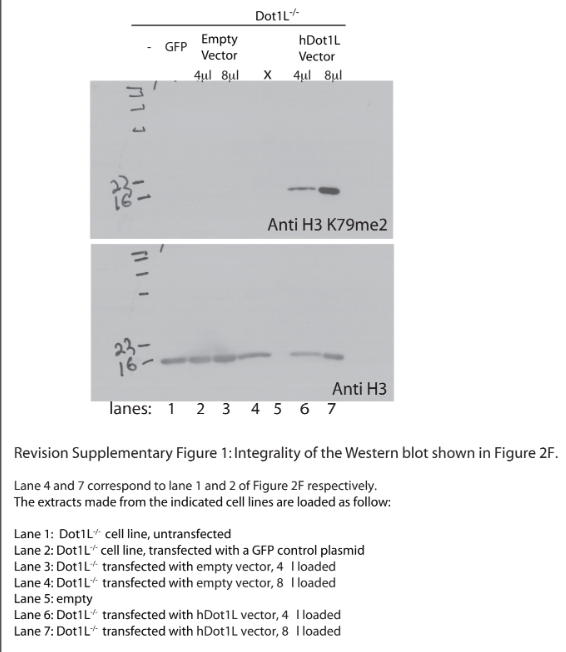

Supplement: S1 File — (DOCX) [file pone.0316233.s001.docx]
